# Treatment patterns of extracorporeal photopheresis in steroid-refractory graft versus host disease: A delphi study

**DOI:** 10.1038/s41409-025-02687-y

**Published:** 2025-07-22

**Authors:** Olaf Penack, Andrea Bacigalupo, Eleni Gavriilaki, Hildegard Greinix, Florent Malard, David Michonneau, Attilio Olivieri, Zinaida Peric, Elisa Sala, Carlos Solano, Daniel Wolff, Robert Zeiser

**Affiliations:** 1 Charité – Universitätsmedizin Berlin, corporate member of Freie Universität Berlin and Humboldt-Universität zu Berlin, Department of Hematology, Oncology and Tumorimmunology, Augustenburger Platz 1, 13353 Berlin, Germany; 2 Fondazione Policlinico Universitario A. Gemelli, Roma, Italy; 3 University of Thessaloniki, Greece; 2nd Propedeutic Department of Internal Medicine, Aristotle University of Thessaloniki, Thessaloniki, Greece; 4 Medizinische Universität Graz, Graz, Austria; 5 Sorbonne Université, Centre de Recherche Saint-Antoine INSERM UMRs938, Service d’Hématologie Clinique et de Thérapie Cellulaire, Hôpital Saint Antoine, AP-HP, Paris, France; 6 Saint Louis Hospital, Paris, France; 7 Università degli Studi di Ancona, Ancona, Italy; 8 University Hospital Centre Rijeka, Rijeka, Croatia; 9 Department of Internal Medicine III, Universitätsklinikum Ulm, Ulm, Germany; 10 Hospital Clínico Universitario-INCLIVA, University of Valencia, Valencia, Spain; 11 Dept. of Medicine III, Universitätsklinikum Regensburg, Regensburg, Germany; 12 Universitätsklinikum Freiburg, Freiburg, Germany

**Keywords:** Bone marrow transplantation, Graft-versus-host disease

Steroid-refractory GvHD (SR-GvHD) following allogeneic transplant is a major clinical challenge and is associated with substantial mortality [1]. While the European Society for Bone and Marrow Transplantation (EBMT) recommends ruxolitinib for second-line treatment of GvHD [2], some patients may experience side effects or refractoriness, and therefore require alternative treatment options [2]. However, there are no standardized treatment recommendations for SR-GvHD beyond ruxolitinib in Europe [2]. Extracorporeal photopheresis (ECP), a cell-based, immunomodulatory therapy, is quoted as a potential treatment option beyond second-line, in the EBMT guidelines [2]. ECP has been shown to be an effective and well-tolerated treatment for SR-GvHD [3]. Clinical evidence has limitations though, including few randomized studies and a lack of consensus on optimal treatment schedule [3, 4]. A Delphi study was thus carried out to provide a descriptive account of clinical practice regarding ECP treatment patterns in SR-GvHD.

Seventeen GvHD experts were invited by Therakos UK Limited (Staines, United Kingdom) to participate in a Delphi panel; of which, 11 agreed. All panelists had extensive experience in GvHD treatment and prescribed ECP or ruxolitinib at their centers.

The Delphi study consisted of three rounds of online questionnaires conducted in 2023 (February 20^th^–March 13^th^; May 31^st^–June 23^rd^; August 4^th^–24^th^), assessing 62 statements. A description of the Delphi questionnaire, the development and scoring processes and a list of the consensus statements are provided in the Appendix. The main findings are summarized in Table 1.

**Table 1:** Summary of key Delphi study results.

Topic	Question to the experts	Results			
		aGvHD	Agreement %	cGvHD	Agreement %
**Reasons for selecting ECP**	Which factors influence you to select ECP as a treatment in SR-GvHD patients?	Rank 1: Efficacy of ECP	100	Rank 1: Efficacy of ECP	100
		Rank 2: Safety profile	100	Rank 2: Safety profile	100
		Rank 3: Steroid-sparing effect	100	Rank 3: Steroid-sparing effect	100
**Combination of ECP with other GvHD therapies**	What are the main reasons for choosing the combination therapy of ECP and ruxolitinib in SR-GvHD patients?	Rank 1: Severe cases	100	Rank 1: Increased efficacy	100
		Rank 2: Increased efficacy	100	Rank 2: Severe cases	100
**Reducing steroid treatment**	Depending on the applied treatment: What is the percentage of SR-GvHD patients where steroids could be reduced by at least 50% Do you agree on the percentages, resulting from round 1?	Proportion of patients treated with ECP: 50%	100	Proportion of patients treated with ECP: 60%	100
		Proportion of patients treated with ruxolitinib: 53%	91	Proportion of patients treated with ruxolitinib: 65%	91
		Proportion of patients treated with ECP-ruxolitinib: 50%	100	Proportion of patients treated with ECP-ruxolitinib: 50%	100
**Stopping steroid treatment**	Depending on the applied treatment: What is the percentage of SR-GvHD patients in your practice where steroid treatment could be stopped completely? Do you agree on these percentages, resulting from round 1?	Proportion of patients treated with ECP: 50%	100	Proportion of patients treated with ECP: 41%	100
		Proportion of patients treated with ruxolitinib: 51%	91	Proportion of patients treated with ruxolitinib: 40%	91
		Proportion of patients treated with ECP-ruxolitinib: 70%	91	Proportion of patients treated with ECP-ruxolitinib: 60%	100
**ECP monotherapy**	Do you agree on the selection criteria for treating SR-GvHD (both acute and chronic) patients with ECP monotherapy?	Rank 1: Low risk (e.g. skin involvement only or upper GI only)		91	
		Rank 2: Contraindication for Ruxolitinib (e.g. thrombocytopenia)		91	
**Treatment duration of ECP/ruxolitinib**	What is the average treatment duration of ECP/ruxolitinib in SR-GvHD in the following scenarios?	ECP: 4 to 6 months	91	ECP: 10 to 12 months	91
		Ruxolitinib: 3 to 5 months	91	Ruxolitinib: 10 to 12 months	100
		ECP in combination with ruxolitinib: 4 to 6 months	100	ECP in combination with ruxolitinib: 8 to 10 months	91
		Ruxolitinib in combination with ECP: 3 to 5 months	91	Ruxolitinib in combination with ECP: 8 to 10 months	91
**Treatment schedules of ECP**	When treating SR-GvHD patients with ECP alone but not with ruxolitinib - which treatment schedules do you apply?	Treatment schedule 1: 2 – 3 ECP procedures on consecutive days weekly for 4 weeks	91	Treatment schedule 1: 2 ECP procedures per week for approximately 9 weeks	55
		Treatment schedule 2: 2 ECP procedures per week at least every two weeks for approximately 8 weeks (2 months)	82	Treatment schedule 2: 2 ECP procedures per week, at least every two weeks for approximately 10 weeks	64
		Treatment schedule 3: 2 ECP procedures per week at least every month for approximately 8 weeks (2 months)	36	Treatment schedule 3: 1 – 2 ECP procedures per week at least monthly for approximately 20 weeks (5 months)	73

*aGvHD* acute graft-versus-host disease, *cGvHD* chronic graft-versus-host disease, *ECP* extracorporeal photopheresis, *GI* gastrointestinal, *SR-GvHD* steroid-refractory graft-versus-host disease.

Experts were asked the main reasons for choosing ECP, but not ruxolitinib, in SR-GvHD. For steroid refractory acute graft-versus-host disease (SR-aGvHD), experts reached consensus (100% agreement for all) on (1) contraindication and safety profile of ruxolitinib (2), ruxolitinib failure, and (3) safety profile and high efficacy of ECP (especially in patients with skin involvement). For steroid refractory chronic graft-versus-host disease (SR-cGvHD), experts reached consensus on (1) contraindication to ruxolitinib (100% agreement) (2), ruxolitinib failure (91% agreement) (3) efficacy, especially in patients with skin involvement and for steroid-sparing effect (91% agreement), and (4) safety profile (100% agreement). Expert consensus aligned with published clinical evidence, which has shown ECP to be well-tolerated and associated with high response rates [3, 5], whereas ruxolitinib, whilst efficacious, has been associated with the incidence of cytopenia and infection [6]. For patients with contraindications to ruxolitinib or who become refractory, ECP may, therefore, provide a suitable alternative.

Experts were asked the main reasons for selecting ruxolitinib, but not ECP, in SR-GvHD. For SR-aGvHD, experts reached consensus on (1) high efficacy, especially in patients with gastrointestinal involvement (100% agreement) (2), regulatory reasons (91% agreement), and (3) venous access not being necessary (91% agreement). For SR-cGvHD, experts reached consensus on (1) high efficacy, especially in patients with gastrointestinal involvement (100% agreement) (2), regulatory reasons (91% agreement) and patient preferences (82% agreement). These results are largely consistent with published literature, which shows ruxolitinib to be highly effective [6], and long-term vascular access, as required by ECP, to be associated with an increased risk of complications (e.g. bacteremia) [7]. Moreover, compared to ruxolitinib, the administration of ECP may be more challenging for patients who live far from ECP centers and for hospitals with limited capacity. However, lack of access to ruxolitinib in some countries such as the UK may limit patient access [8]. In which case, ECP may provide an alternative treatment option for these patients.

Expert consensus for the definition of ruxolitinib refractoriness (Appendix Table 2), a common challenge during GvHD treatment [9, 10], was reached and broadly aligned with the criteria proposed by Mohty et al. for aGvHD [11]. For cGvHD, the following definition reached consensus: progression of GvHD after 1–2 weeks of treatment, lack of improvement in GvHD after 2–3 months of treatment and loss of response, defined as objective worsening of GvHD. Given the importance of ruxolitinib refractoriness as a selection factor and limited literature on the positioning of ECP following EBMT’s ruxolitinib recommendation, the Delphi study’s proposed definition of ruxolitinib refractoriness offers an opportunity for standardization of the rationale for selecting ECP over ruxolitinib.

Experts agreed that there were no obstacles to combining ECP with any other guideline-recommended therapy for aGvHD (100%) and cGvHD (91%), respectively. The clinical evidence for combination therapy is, however, limited to retrospective studies on combined ECP and ruxolitinib treatment [3]. Future research on ECP combination therapy may, therefore, offer valuable insights into its use in SR-GvHD and expand treatment options for patients. In line with this, ECP combination therapy in GvHD was identified as a research priority in the 2024 EBMT guidelines [2].

All experts agreed that, in 50% of aGvHD and 60% of cGvHD patients treated with ECP, steroids could be reduced by ≥50%. All experts agreed that in 80% of aGvHD and cGvHD patients treated with the combination of ECP and ruxolitinib, steroids could also be reduced by ≥50%. 91% of experts agreed that an average of ~29 days and ~56 days is required to implement a 50% steroid reduction in aGvHD and cGvHD patients treated with ECP, respectively. All experts agreed that steroid treatment could be stopped in 50% of aGvHD and ~40% of cGvHD patients treated with ECP. All experts agreed that an average of 2–3 months and 5.5 months is required to stop steroid treatment in aGvHD and cGvHD patients treated with ECP, respectively. This potential steroid-sparing effect of ECP, as evidenced in multiple studies [3], may help circumvent the significant toxicity and mortality associated with long-term steroid use, notably in cGvHD [12], and improve patients’ health-related quality of life.

Experts were asked to estimate the average treatment duration and frequency of ECP in SR-GvHD but reached partial and no consensus for SR-aGvHD and SR-cGvHD, respectively. For SR-aGvHD, 91% of experts agreed that the average duration of ECP is approximately 4 to 6 months, while the highest level of agreement (91%) for treatment frequency was 2 to 3 ECP procedures on consecutive days weekly for 4 weeks. Published ECP treatment recommendations for SR-aGvHD mostly suggest weekly treatment with varying tapering regimens [4]. For SR-cGvHD, the highest level of agreement for treatment duration was reached for 10 to 12 months (91%), and for a treatment frequency of 1 to 2 ECP procedures per week at least monthly for approximately 20 weeks (73%). ECP treatment recommendations for SR-cGvHD in the literature are more varied than SR-aGvHD, with studies suggesting weekly and biweekly treatment regimens [4]. This variation may be due to patient-specific factors or differences in center-specific immunosuppression regimens [4].

This Delphi study combined the views of multiple international experts in the field of GvHD and use of ECP, and offers insights into real-world ECP treatment patterns. However, expert opinions are subject to their respective clinical experience, which may not reflect country-wide clinical practice. While the selection of experts with experience in ECP by Therakos ensured that the results were valid, the small sample size (n = 11) may not reflect country-specific nuances and resources which may influence treatment patterns. The results should therefore be interpreted with these limitations in mind.

## Supplementary information

ECP for GvHD Manuscript_Supplemental Files
