# Supplementary material for: Treatment patterns of extracorporeal photopheresis in steroid-refractory graft versus host disease: A delphi study
Source: Bone Marrow Transplant. 2025 Jul 22;60(10):1398–401. doi: 10.1038/s41409-025-02687-y (PMC12568635; doi:10.1038/s41409-025-02687-y)
Supplement: Supplementary file 1 — ECP for GvHD Manuscript_Supplemental Files [file 41409_2025_2687_MOESM1_ESM.pdf]

SUPPLEMENTARY INFORMATION

Description of the Delphi method

These questions were developed based on current literature, including gaps in literature, and input from a SR-GvHD clinical expert (**OP**). The question types included open-ended, nominal, ratio, ranking, binary (yes-no), and ordinal questions with a 5-point Likert scale ("strongly disagree", "disagree", "neutral", "agree" and "strongly agree"). Consensus statements for round 2 were refined based on aggregated round 1 answers and included the numerical values provided by experts in round 1. Consensus was reached if ≥75% of experts agreed (defined as "agree" or "totally agree") with the statements. For statements which did not reach consensus in round 2, the statements were adapted and presented in round 3.

Delphi study round 1 questionnaire

Thank you for participating in this survey.

The context of this survey is the growing availability of treatments for steroid refractory acute graft-versus-host disease (aGvHD) in grades II-IV and moderate-severe chronic graft-versus-host disease(cGvHD) following HSCT.

We are keen on understanding the evolving practices specifically in regards to the prescription of extracorporeal photopheresis (ECP) in the real world.

The main objectives of the survey are firstly to gain a better

understanding of clinicians' decision-making process that led to prescribing ECP in the context of new available treatments. Secondly, the objective is to understand timing and frequency of ECP treatments in the new prescribing environment.

Survey methodology: Delphi panel

Once you have answered all questions, your answers will be anonymised and aggregated with the answers from other European participants. All responses will be compiled into a new questionnaire for you to review and comment on in the second round. Finally, the outputs of the second round will be presented to you and the other participants of the Delphi panel for final review and comments.

We would like to ask you to answer the survey based on your own experience. We estimate that it will take 120 minutes to complete the initial survey. Please allow enough time to be able to complete all questions. We would like to thank you in advance for your contribution to this survey.

Your study team

This study is sponsored by Therakos UK Limited.

I. Treatment of steroid refractory GvHD with ECP

Q1.1: What are your top treatment goals in treating steroid refractory aGvHD? Please rank the following goals according to their relative importance:

- Improve length of survival __________
- Achieve partial response __________
- Achieve complete response __________
- Improve quality of life __________
- Another goal, namely: __________

Q1.2: What are your top treatment goals in treating steroid refractory cGvHD? Please rank the following goals according to their relative importance:

- Improve length of survival __________
- Achieve partial response __________
- Achieve complete response __________
- Improve quality of life __________
- Another goal, namely: __________

Q2.1: Which factors influence you to select ECP as a treatment in steroid refractory aGvHD patients? Please rank the following factors according to their relative importance:

- Efficacy of ECP __________
- Possibility to combine with other immunosuppressive therapies __________
- Safety profile __________
- Steroid-sparing effect __________
- Patients' adherence to treatment __________
- Another factor, namely: __________

Q2.2: Which factors influence you to select ECP as a treatment in steroid refractory cGvHD patients? Please rank the following

factors according to their relative importance:

- Efficacy of ECP __________
- Possibility to combine with other immunosuppressive therapies __________
- Safety profile __________
- Steroid-sparing effect __________
- Patients' adherence to treatment __________
- Another factor, namely: __________

Q3.1: When treating steroid refractory aGvHD patients with ECP alone* but not with ruxolitinib - which treatment schedules do you apply?

* +/- steroids and/or CNI

Example: You treat patients in three phases: Initiation phase, maintenance phase, and tapering phase. In the initiation phase, you apply 3 ECPs procedures on consecutive days weekly for 8 weeks; in the maintenance phase you apply 2 ECPs on consecutive days weekly for 24 weeks; in the tapering phase you apply 1 ECP bi-weekly for 52 weeks.

	Number of ECP procedures
Treatment schedule 1	
Treatment schedule 2	
Treatment schedule 3	

Q3.1: When treating steroid refractory aGvHD patients with ECP alone* but not with ruxolitinib - which treatment schedules do

you apply?

* +/- steroids and/or CNI

Example: You treat patients in three phases: Initiation phase, maintenance phase, and tapering phase. In the initiation phase, you apply 3 ECPs procedures on consecutive days weekly for 8 weeks; in the maintenance phase you apply 2 ECPs on consecutive days weekly for 24 weeks; in the tapering phase you apply 1 ECP bi-weekly for 52 weeks.

	Consecutive days
Treatment schedule 1	yes<br>no
Treatment schedule 2	yes<br>no
Treatment schedule 3	yes<br>no

Q3.1: When treating steroid refractory aGvHD patients with ECP alone* but not with ruxolitinib - which treatment schedules do you apply?

* +/- steroids and/or CNI

Example: You treat patients in three phases: Initiation phase, maintenance phase, and tapering phase. In the initiation phase, you apply 3 ECPs procedures on consecutive days weekly for 8 weeks; in the maintenance phase you apply 2 ECPs on consecutive days weekly for 24 weeks; in the tapering phase you apply 1 ECP bi-weekly for 52 weeks.

	Frequency
Treatment schedule 1	weekly<br>every two weeks<br>monthly<br>twice a month
Treatment schedule 2	weekly<br>every two weeks<br>monthly<br>twice a month
Treatment schedule 3	weekly<br>every two weeks<br>monthly<br>twice a month

Q3.1: When treating steroid refractory aGvHD patients with ECP alone* but not with ruxolitinib - which treatment schedules do you apply?

* +/- steroids and/or CNI

Example: You treat patients in three phases: Initiation phase, maintenance phase, and tapering phase.In the initiation phase, you apply 3 ECPs procedures on consecutive days weekly for 8 weeks; in the maintenance phase you apply 2 ECPs on consecutive days weekly for 24 weeks;in the tapering phase you apply 1 ECP bi-weekly for 52 weeks.

	Duration (in weeks)
Treatment schedule 1	
Treatment schedule 2	
Treatment schedule 3	

Q3.2: When treating steroid refractory cGvHD patients with ECP alone* - which treatment schedules do you apply?

*+/- steroids and/or CNI

	Number of ECP procedures
Treatment schedule 1	
Treatment schedule 2	
Treatment schedule 3	

Q3.2: When treating steroid refractory cGvHD patients with ECP alone* - which treatment schedules do you apply?

*+/- steroids and/or CNI

	Consecutive days
Treatment schedule 1	yes<br>no
Treatment schedule 2	yes<br>no
Treatment schedule 3	yes<br>no

Q3.2: When treating steroid refractory cGvHD patients with ECP alone* - which treatment schedules do you apply?*+/- steroids and/or CNI

	Frequency
Treatment schedule 1	weekly<br>every two weeks<br>monthly<br>twice a month
Treatment schedule 2	weekly<br>every two weeks<br>monthly<br>twice a month
Treatment schedule 3	weekly<br>every two weeks<br>monthly<br>twice a month

Q3.2: When treating steroid refractory cGvHD patients with ECP alone* - which treatment schedules do you apply?*+/- steroids and/or CNI

	Duration (in weeks)
Treatment schedule 1	
Treatment schedule 2	
Treatment schedule 3	

Q4.1: Would you ever continue ECP treatment despite a lack of visible clinical improvements?

1. Yes
2. No

Q4.2: What are your reasons for continuing ECP treatments? Please rank the following factors according to their relative importance:

- Possibility to combine ECP with other immunosuppressive treatment __________
- ECP's safety profile __________
- Improvement of quality of life despite clinical improvements __________
- Patients' preferences __________
- Other reasons, namely: __________

Q5: In which organs do you see the most improvements following ECP treatment? Please drag the organs with the most improvements to the top of the list in the white box and the organs with the least improvements to the bottom of the list.

- Eyes __________
- Fascia __________
- Genital mucosa __________
- Gut __________
- Kidney __________
- Liver __________
- Lung __________
- Musculosceletal __________

- Oral mucosa __________
- Salivary glands __________
- Skin __________
- Other __________

Thank you, you have reached the end of the first section.

II. Patient numbers and treatment duration

Q6.1: In your own practice, what is the percentage of patients with steroid refractory aGvHD that receive various treatments as per described below?

- Regimen containing neither ruxolitinib nor ECP __________
- Regimen containing ruxolitinib, but no ECP __________
- Regimen containing ECP, but no ruxolitinib __________
- Regimen containing both, ruxolitinib and ECP __________

Q6.2: In your own practice, what is the percentage of patients with steroid refractory cGvHD that receive various treatments as per described below?

- Regimen containing neither ruxolitinib nor ECP __________
- Regimen containing ruxolitinib, but no ECP __________
- Regimen containing ECP, but no ruxolitinib __________
- Regimen containing both, ruxolitinib and ECP __________

Q6.3: Of the steroid refractory aGvHD patients receiving a combination therapy with ECP and ruxolitinib - what percentage start both treatments simultaneously?

Percentage of initial ECP-ruxolitinib combinations on all ECP-ruxolitinib combinations	❑

Q6.4: Of the steroid refractory cGvHD patients receiving a combination therapy with ECP and ruxolitinib- what percentage start both treatments simultaneously?

Percentage of initial ECP-ruxolitinib combinations on all ECP-ruxolitinib combinations	❑

Q7: What is your main reason for choosing the treatments below?

	aGvHD	cGvHD
ECP but no ruxolitinib	❑	❑
Ruxolitinib but no ECP	❑	❑
ECP + ruxolitinib together	❑	❑

Q8: Based on your practice, what is the average time between steroid refractory diagnosis and treatment start for the treatments below? Please indicate whether your answer refers to days, weeks or months.

	aGvHD Time
ECP but no ruxolitinib	
Ruxolitinib but no ECP	
Combination treatment(ECP + ruxolitinib)	

Q8: Based on your practice, what is the average time between steroid refractory diagnosis and treatment start for the treatments below? Please indicate whether your answer refers to days, weeks or months.

	aGvHD Unit
ECP but no ruxolitinib	Days<br>Weeks<br>Months
Ruxolitinib but no ECP	Days<br>Weeks<br>Months
Combination treatment (ECP + ruxolitinib)	Days<br>Weeks<br>Months

Q8: Based on your practice, what is the average time between steroid refractory diagnosis and treatment start for the

treatments below? Please indicate whether your answer refers to days, weeks or months.

	cGvHD Time
ECP but no ruxolitinib	
Ruxolitinib but no ECP	
Combination treatment(ECP + ruxolitinib)	

Q8: Based on your practice, what is the average time between steroid refractory diagnosis and treatment start for the treatments below? Please indicate whether your answer refers to days, weeks or months.

	cGvHD Unit
ECP but no ruxolitinib	Days<br>Weeks<br>Months
Ruxolitinib but no ECP	Days<br>Weeks<br>Months
Combination treatment (ECP + ruxolitinib)	Days<br>Weeks<br>Months

Q9.1: Based on your practice and depending on the applied treatment what is the average treatment duration of ECP/ruxolitinib in steroid refractory aGvHD in the following scenarios? Please indicate whether your answer refers to days,

weeks or months.

	Average treatment duration
ECP without ruxolitinib	
Ruxolitinib without ECP	
ECP in combination with ruxolitinib	
Ruxolitinib in combination with ECP	

Q9.1: Based on your practice and depending on the applied treatment what is the average treatment duration of ECP/ruxolitinib in steroid refractory aGvHD in the following scenarios? Please indicate whether your answer refers to days, weeks or months.

	Unit
ECP without ruxolitinib	Days<br>Weeks<br>Months
Ruxolitinib without ECP	Days<br>Weeks<br>Months
ECP in combination with ruxolitinib	Days<br>Weeks<br>Months
Ruxolitinib in combination with ECP	Days<br>Weeks<br>Months

Q9.2: Based on your practice and depending on the applied treatment, what is the average treatment duration of ECP/ruxolitinib in steroid refractory cGvHD in the following scenarios? Please indicate whether your answer refers to days, weeks or months.

	Average treatment duration
ECP without ruxolitinib	
Ruxolitinib without ECP	
ECP in combination with ruxolitinib	
Ruxolitinib in combination with ECP	

Q9.2: Based on your practice and depending on the applied treatment, what is the average treatment duration of ECP/ruxolitinib in steroid refractory cGvHD in the following scenarios? Please indicate whether your answer refers to days, weeks or months.

	Unit
ECP without ruxolitinib	Days<br>Weeks<br>Months
Ruxolitinib without ECP	Days<br>Weeks<br>Months
ECP in combination with ruxolitinib	Days

	Weeks<br>Months
Ruxolitinib in combination with ECP	Days<br>Weeks<br>Months

Q10.1: What percentage of patients that are treated with ECP switch from ECP to ruxolitinib?

aGvHD	❑
cGvHD	❑

Q10.2: What percentage of patients that are treated with ruxolitinib switch from ruxolitinib to ECP?

aGvHD	❑
cGvHD	❑

Q10.3: What percentage of patients that are treated with ECP switch from ECP to a combination therapy of ECP and ruxolitinib?

aGvHD	❑
cGvHD	❑

Q10.4: What percentage of patients that are treated with ruxolitinib switch from ruxolitinib to a combination therapy of ECP and ruxolitinib?

aGvHD	❑
cGvHD	❑

Thank you, you have reached the end of the second section.

III. Combination and Tapering

Q11.1: Depending on the applied treatment: In your practice, what is the percentage of steroid refractory GvHD patients where steroids could be reduced by at least 50%?

	aGvHD
ECP but no ruxolitinib	
Ruxolitinib but no ECP	
Combination treatment (ECP +ruxolitinib)	

Thank you, you have reached the end of the second section.

IV. Combination and Tapering

Q11.1: Depending on the applied treatment: In your practice, what is the percentage of steroid refractory GvHD patients where steroids could be reduced by at least 50%?

	cGvHD
ECP but no ruxolitinib	
Ruxolitinib but no ECP	
Combination treatment (ECP +ruxolitinib)	

Q11.2: How long would it take before you would be able to implement a 50% reduction of steroid dosage? Please indicate whether your answer refers to days, weeks, months.

	aGvHD Time
ECP but no ruxolitinib	
Ruxolitinib but no ECP	
Combination treatment (ECP +ruxolitinib)	

Q11.2: How long would it take before you would be able to implement a 50% reduction of steroid dosage? Please indicate whether your answer refers to days, weeks, months.

	aGvHD Unit
ECP but no ruxolitinib	Days

	Weeks<br>Months
Ruxolitinib but no ECP	Days<br>Weeks<br>Months
Combination treatment (ECP +ruxolitinib)	Days<br>Weeks<br>Months

Q11.2: How long would it take before you would be able to implement a 50% reduction of steroid dosage? Please indicate whether your answer refers to days, weeks, months.

	cGvHD Time
ECP but no ruxolitinib	
Ruxolitinib but no ECP	
Combination treatment (ECP +ruxolitinib)	

Q11.2: How long would it take before you would be able to implement a 50% reduction of steroid dosage? Please indicate whether your answer refers to days, weeks, months.

	cGvHD Unit
ECP but no ruxolitinib	Days<br>Weeks<br>Months
Ruxolitinib but no ECP	Days

	Weeks<br>Months
Combination treatment (ECP +ruxolitinib)	Days<br>Weeks<br>Months

Q11.3: Depending on the applied treatment: What is the percentage of steroid refractory GvHD patients in your practice where steroid treatment could be stopped completely?

	aGvHD
ECP but no ruxolitinib	
Ruxolitinib but no ECP	
Combination treatment (ECP +ruxolitinib)	

Q11.3: Depending on the applied treatment: What is the percentage of steroid refractory GvHD patients in your practice where steroid treatment could be stopped completely?

	cGvHD
ECP but no ruxolitinib	
Ruxolitinib but no ECP	
Combination treatment(ECP +ruxolitinib)	

Q11.4: How long would it take before you would be able to stop steroid treatment completely? Please indicate whether your answer refers to days, weeks, months.

	aGvHD Time
ECP but no ruxolitinib	
Ruxolitinib but no ECP	
Combination treatment(ECP +ruxolitinib)	

Q11.4: How long would it take before you would be able to stop steroid treatment completely? Please indicate whether your answer refers to days, weeks, months.

	aGvHD Unit
ECP but no ruxolitinib	Days<br>Weeks<br>Months
Ruxolitinib but no ECP	Days<br>Weeks<br>Months
Combination treatment (ECP +ruxolitinib)	Days<br>Weeks<br>Months

Q11.4: How long would it take before you would be able to stop steroid treatment completely? Please indicate whether your answer refers to days, weeks, months.

	cGvHD Time
ECP but no ruxolitinib	
Ruxolitinib but no ECP	
Combination treatment(ECP +ruxolitinib)	

Q11.4: How long would it take before you would be able to stop steroid treatment completely? Please indicate whether your answer refers to days, weeks, months.

	cGvHD Unit
ECP but no ruxolitinib	Days<br>Weeks<br>Months
Ruxolitinib but no ECP	Days<br>Weeks<br>Months
Combination treatment(ECP +ruxolitinib)	Days<br>Weeks<br>Months

Q12.1: How do you determine that an aGvHD patient is refractory to ruxolitinib? Please rank the following criteria according to their relative importance:

- Progression in any organ within 3, 4 or 5 days of therapy onset with ruxolitinib __________
- Failure to improve within 5 to 7 days of treatment initiation __________
- Incomplete response after more than 28 days of

ruxolitinib __________

- I apply the definition suggested by Mothy et al, 2020 (please click on question mark icon on the question above to see the definition) __________
- I use a different definition for ruxolitinib-refractory aGVHD (please indicate) __________

Q12.2: How do you determine that a cGvHD patient is refractory to ruxolitinib? Please rank the following criteria according to their relative importance:

- Progression of GVHD while on ruxolitinib for 1-2 weeks __________
- Stable GVHD while on ruxolitinib for 1-2 months __________
- I use a different definition for ruxolitinib-refractory cGVHD (please indicate) __________

Q12.3: In your clinical practice, what is the average treatment time on ruxolitinib before ruxolitinib refractoriness is established? Please indicate whether your answer refers to days, weeks, months or years.

	aGvHD Time
Time period to establish refractoriness	

Q12.3: In your clinical practice, what is the average treatment

time on ruxolitinib before ruxolitinib refractoriness is established? Please indicate whether your answer refers to days, weeks, months or years.

	aGvHD Unit
Time period to establish refractoriness	Days<br>Weeks<br>Months

Q12.3: In your clinical practice, what is the average treatment time on ruxolitinib before ruxolitinib refractoriness is established? Please indicate whether your answer refers to days, weeks, months or years.

	cGvHD Time
Time period to establish refractoriness	

Q12.3: In your clinical practice, what is the average treatment time on ruxolitinib before ruxolitinib refractoriness is established? Please indicate whether your answer refers to days, weeks, months or years.

	cGvHD Unit
Time period to establish refractoriness	Days<br>Weeks<br>Months

Q13.1: When treating steroid refractory aGvHD patients with

ECP, what would be reasons for stopping ECP treatment?

- Inconvenience for the patient __________
- Lack of efficacy __________
- Side effects __________
- Another reason, namely: __________

Q13.2: When treating steroid refractory cGvHD patients with ECP, what would be reasons for stopping ECP treatment?

- Inconvenience for the patient __________
- Lack of efficacy __________
- Side effects __________
- Another reason, namely: __________

Q14.1: When treating steroid refractory aGvHD patients with ruxolitinib, what would be reasons for stopping ruxolitinib treatment?

- Inconvenience for the patient __________
- Lack of efficacy __________
- Side effects __________
- Another reason, namely: __________

Q14.2: When treating steroid refractory cGvHD patients with ruxolitinib, what would be the reasons for stopping ruxolitinib treatment?

- Inconvenience for the patient __________
- Lack of efficacy __________
- Side effects __________
- Another reason, namely: __________

Q15.1: When treating steroid refractory aGvHD patients with combination therapy (ECP & ruxolitinib), what would be reasons to stop the combination treatment?

- Inconvenience for the patient __________
- Lack of efficacy __________
- Side effects __________
- Another reason, namely: __________

Q15.2: When treating steroid refractory cGvHD patients with combination treatment (ECP & ruxolitinib), what would be reasons to stop the combination treatment?

- Inconvenience for the patient __________
- Lack of efficacy __________
- Side effects __________
- Another reason, namely: __________

Q16.1: Based on your experience please list the reasons you would NOT add ECP to ruxolitinib in steroid refractory aGvHD patients. Please order the reasons from most important to least important.

- Low efficacy __________
- Inconvenience for the patient __________
- High workload for the hospital staff __________
- ECP capacity in my center is restricted __________
- Other reason, please specify __________

Q16.2: Based on your experience please list the reasons you

would NOT add ECP to ruxolitinib in steroid refractory cGvHD patients. Please order the reasons from most important to least important.

- Low efficacy __________
- Inconvenience for the patient __________
- High workload for the hospital staff __________
- ECP capacity in my center is restricted __________
- Other reason, please specify __________

Q17: Based on your experience and depending on the treatment schemes: When do you decide to add an additional treatment to the initial therapy? Please indicate whether your answer refers to days, weeks, or months.

	aGvHD Time
Average time period from ruxolitinib to addition of ECP	
Average time period from ECP to addition of ruxolitinib	

Q17: Based on your experience and depending on the treatment schemes: When do you decide to add an additional treatment to the initial therapy? Please indicate whether your answer refers to days, weeks, or months.

	aGvHD Unit
Average time period from ruxolitinib to	Days

addition of ECP	Weeks<br>Months
Average time period from ECP to addition of ruxolitinib	Days<br>Weeks<br>Months

Q17: Based on your experience and depending on the treatment schemes: When do you decide to add an additional treatment to the initial therapy? Please indicate whether your answer refers to days, weeks, or months.

	cGvHD Time
Average time period from ruxolitinib to addition of ECP	
Average time period from ECP to addition of ruxolitinib	

Q17: Based on your experience and depending on the treatment schemes: When do you decide to add an additional treatment to the initial therapy? Please indicate whether your answer refers to days, weeks, or months.

	cGvHD Unit
Average time period from ruxolitinib to addition of ECP	Days<br>Weeks<br>Months
Average time period from ECP to addition of ruxolitinib	Days<br>Weeks

	Months

Q18: Please list below the advantages/ of using a sequential treatment regimen of ECP and ruxolitinib.

	aGvHD	cGvHD
Advantage 1	❑	❑
Advantage 2	❑	❑
Advantage 3	❑	❑

Q19.1: In your experience, would it be possible to taper the dosage of ruxolitinib as part of a combination therapy with ECP at all?

	aGvHD
Ruxolitinib tapering possible	Yes<br>No

Q19.1: In your experience, would it be possible to taper the dosage of ruxolitinib as part of a combination therapy with ECP at all?

	cGvHD
Ruxolitinib tapering possible	Yes<br>No

Q19.2: In your experience, would it be possible to taper the ECP treatment schedule as part of a combination therapy with ruxolitinib at all?

	aGvHD
ECP tapering possible	Yes<br>No

Q19.2: In your experience, would it be possible to taper the ECP treatment schedule as part of a combination therapy with ruxolitinib at all?

	cGvHD
ECP tapering possible	Yes<br>No

Q19.3: If steroid refractory GvHD patients respond to the combination therapy of ECP and ruxolitinib you may want to reduce treatment. How would you design the reduction of combination therapy?

	aGvHD
Reduction of ruxolitinib first	❍
Reduction of ECP first	❍

Simultaneous reduction of ruxolitinib and ECP	❍

Q19.3: If steroid refractory GvHD patients respond to the combination therapy of ECP and ruxolitinib you may want to reduce treatment. How would you design the reduction of combination therapy?

	cGvHD
Reduction of ruxolitinib first	❍
Reduction of ECP first	❍
Simultaneous reduction of ruxolitinib and ECP	❍

Q20: Is the ECP treatment schedule adapted when used in combination? If yes, please describe the adaption.

	aGvHD Adaption [yes/no]
	Yes<br>No

Q20: Is the ECP treatment schedule adapted when used in combination? If yes, please describe the adaption.

	aGvHD If yes,

	description of adaption

Q20: Is the ECP treatment schedule adapted when used in combination? If yes, please describe the adaption.

	cGvHD Adaption [yes/no]
	Yes<br>No

Q20: Is the ECP treatment schedule adapted when used in combination? If yes, please describe the adaption.

	cGvHD If yes, description of adaption

Q21: Please list the 3 therapies you are most likely to combine with ECP.

	aGvHD	cGvHD
Therapy 1	❑	❑
Therapy 2	❑	❑

Therapy 3	❑	❑

Q22: Based on your experience, what would be the hurdles preventing a timely use of ECP after steroid refractoriness has been established? Please rank the following reasons from most important to least important.

- Restricted capacity of the ECP unit __________
- Restricted hospital bed capacity __________
- Difficulties with venous access for ECP __________
- Patient decision __________
- Different reason/hurdle: (please indicate) __________

Thank you, you have reached the end of the third section.

V. Exploratory questions on ECP

Q23: Do you see the potential of using ECP as a monotherapy (without steroids) in GvHD patients? Please note: For this question you can also consider low grade or mild GvHD patients.

	Yes	No
aGvHD	❑	❑
cGvHD	❑	❑

Q24: What percentage of GvHD patients would you consider treating with ECP even if they are not refractory to steroids?

aGvHD	❏
cGvHD	❏

Q25: You have now reached the end of the questionnaire. Are there any other aspects related to ECP treatment of steroid-refractory GvHD patients that are of high importance to you and were not addressed in the questionnaire?

Delphi study round 2 questionnaire

Dear participant,

welcome to the second round of the Delphi Panel on the use of extracorporeal photopheresis (ECP) in steroid refractory acute graft-versus-host disease (aGvHD) in grades II-IV and moderate-severe chronic graft-versus-host disease (cGvHD) following HSCT.

We would like to thank you for responding to the first questionnaire.

We evaluated and summarized the results of the first round to develop this new version. The aim of this second round of questioning is to vote on the results of the first round and ideally to reach a consensus.

Please allow sufficient time to complete the questionnaire. The estimated time required is 60-120 minutes. Your participation in this study will remain anonymous to the other participants.

We would like to thank you in advance for your participation and support!

Your study team

IV. Treatment of steroid refractory GvHD with ECPQ1.1: What are your top treatment goals in treating steroid refractory aGvHD? Do you agree on the ranking, resulting from round 1?

	Strongly disagree	Disagree	Neither nor	Agree	Strongly agree
Rank 1 (most important): Achieve complete response	❑	❑	❑	❑	❑
Rank 2: Improve length of survival	❑	❑	❑	❑	❑

Rank 3 (least important): Achieve partial response	❑	❑	❑	❑	❑

You disagreed on the ranking from round 1. What are your top treatment goals in treating steroid refractory aGvHD? Please rank the following goals according to their relative importance:

- Improve length of survival __________
- Achieve partial response __________
- Achieve complete response __________
- Improve quality of life __________
- Another goal, namely: __________

Q1.2: What are your top treatment goals in treating steroid refractory cGvHD? Do you agree on the ranking, resulting from round 1?

	Strongly disagree	Disagree	Neither nor	Agree	Strongly agree
Rank 1 (most important): Achieve complete response	❑	❑	❑	❑	❑
Rank 2: Improve length of survival	❑	❑	❑	❑	❑
Rank 3 (least important): Improve quality of life	❑	❑	❑	❑	❑

You disagreed with the ranking from round 1. What are your top treatment goals in treating steroid refractory cGvHD? Please rank the following goals according to their relative importance:

- Improve length of survival __________
- Achieve partial response __________
- Achieve complete response __________
- Improve quality of life __________
- Another goal, namely: __________

Q2.1:Which factors influence you to select ECP as a treatment in steroid refractory aGvHD patients? Do you agree on the ranking, resulting from round 1?

	Strongly disagree	Disagree	Neither nor	Agree	Strongly agree
Rank 1 (most important): Efficacy of ECP	❑	❑	❑	❑	❑
Rank 2: Safety profile	❑	❑	❑	❑	❑
Rank 3 (least important): Steroid-sparing effect	❑	❑	❑	❑	❑

You disagreed on the ranking from round 1. Which factors

influence you to select ECP as a treatment in steroid refractory aGvHD patients? Please rank the following factors according to their relative importance:

- Efficacy of ECP __________
- Possibility to combine with other immunosuppressive therapies __________
- Safety profile __________
- Steroid-sparing effect __________
- Patients' adherence to treatment __________
- Another factor, namely: __________

Q2.2: Which factors influence you to select ECP as a treatment in steroid refractory cGvHD patients? Do you agree on the ranking, resulting from round 1?

	Strongly disagree	Disagree	Neither nor	Agree	Strongly agree
Rank 1 (most important): Efficacy of ECP	❑	❑	❑	❑	❑
Rank 2: Safety profile	❑	❑	❑	❑	❑
Rank 3 (least important): Steroid-sparing effect	❑	❑	❑	❑	❑

You disagreed on the ranking from round 1. Which factors influence you to select ECP as a treatment in steroid

refractory cGvHD patients? Please rank the following factors according to their relative importance:

- Efficacy of ECP __________
- Possibility to combine with other immunosuppressive therapies __________
- Safety profile __________
- Steroid-sparing effect __________
- Patients’ adherence to treatment __________
- Another factor, namely: __________

Q3.1: When treating steroid refractory aGvHD patients with ECP alone* but not with ruxolitinib – which treatment schedules do you apply?

* +/- steroids and/or CNI.

Do you agree on these statements, resulting from round 1?

	Strongly disagree	Disagree	Neither nor	Agree	Strongly agree
Treatment schedule 1: 2-3 ECP procedures per week on consecutive days weekly for 4 weeks	❑	❑	❑	❑	❑
Treatment schedule 2: 1-2 ECP procedure per week on consecutive days weekly for 8 weeks	❑	❑	❑	❑	❑
Treatment schedule 3: 1-2 ECP procedures per week on consecutive days monthly for 8 weeks	❑	❑	❑	❑	❑

You disagreed on the statement. When treating steroid refractory aGvHD patients with ECP alone* - which treatment schedules (1-3) do you apply?

*+/- steroids and/or CNI

Q3.2: When treating steroid refractory cGvHD patients with ECP alone* - which treatment schedules do you apply?

*+/- steroids and/or CNI.

Do you agree on these statements, resulting from round 1?

	Strongly disagree	Disagree	Neither nor	Agree	Strongly agree
Treatment schedule 1: 2 ECP procedures per week on consecutive days weekly for 9 weeks.	❑	❑	❑	❑	❑
Treatment schedule 2: 1-2 ECP procedures per week on consecutive days weekly for 10 weeks.	❑	❑	❑	❑	❑
Treatment schedule 3: 1-2 ECP procedures per week on consecutive days monthly for 5 months.	❑	❑	❑	❑	❑

You disagreed on the statement. When treating steroid refractory cGvHD patients with ECP alone* - which treatment schedules (1-3) do you apply?

*+/- steroids and/or CNI

Q4: In which organs do you see the most improvements following ECP treatment? Do you agree on the organ ranking below, resulting from round 1?

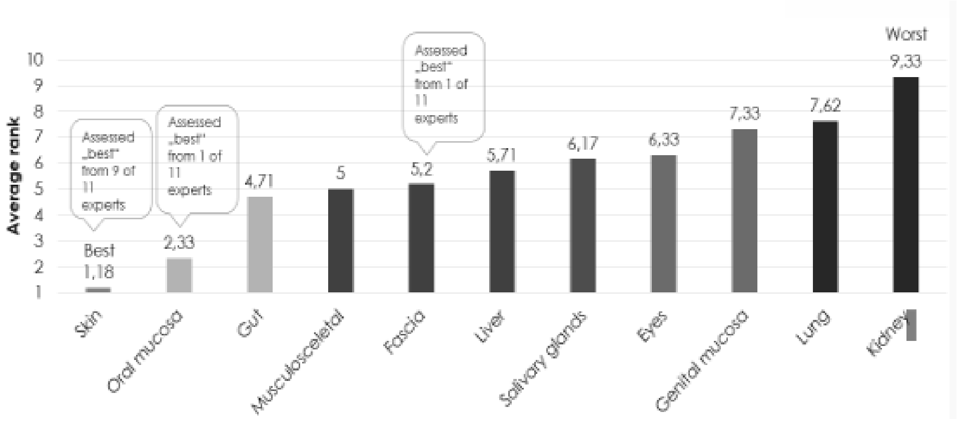

	Strongly disagr	Disagree	Neither nor	Agree	Strongly agree

	ee				
Rank 1 (Most improvements): Skin	❑	❑	❑	❑	❑
Rank 2: Oral mucosa	❑	❑	❑	❑	❑
Rank 3: Gut, musculoskeletal, fascia, liver, salivary glands, eyes, genital mucosa and lung	❑	❑	❑	❑	❑
Rank 4 (Least improvements): Kidney	❑	❑	❑	❑	❑

You disagreed on the ranking. In which organs do you see the most improvements following ECP treatment? Please rank the following organs according to 1 to 9. 1 most improvements to 9; least improvements.

- Skin __________
- Oral mucosa __________
- Gut __________
- Musculoskeletal fascia __________
- Liver __________
- Salivary glands __________
- Eyes __________
- Genital mucosa __________
- Lung __________

Thank you, you have reached the end of the first section.

II. Patient numbers and treatment duration

Q5.1: What is the percentage of patients with steroid refractory aGvHD that receive various treatments as per described below?

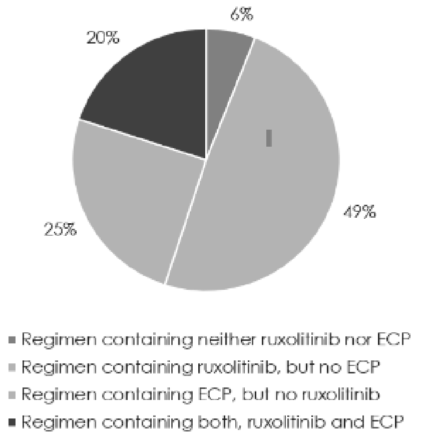

Do you agree on these percentages, resulting from round 1?

	Strongly disagree	Disagree	Neither nor	Agree	Strongly agree
Approx. 49% of aGvHD patients receive a regimen containing ruxolitinib, but no ECP.	❑	❑	❑	❑	❑
Approx. 25% of aGvHD patients receive a regimen containing ECP, but no ruxolitinib.	❑	❑	❑	❑	❑
Approx. 20% of aGvHD patients receive a regimen	❑	❑	❑	❑	❑

containing both ECP and ruxolitinib.					
Approx. 6% of aGvHD patients receive neither ECP nor ruxolitinib.	❑	❑	❑	❑	❑

Q5.2: What is the percentage of patients with steroid refractory cGvHD that receive various treatments as per described below?

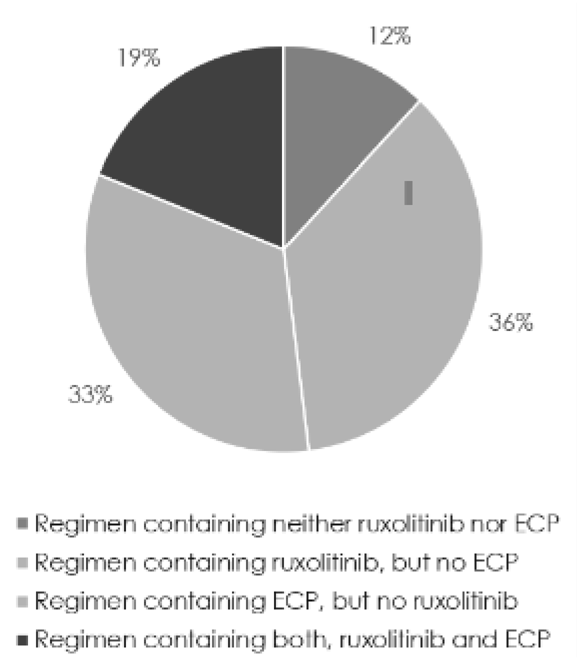

Do you agree on these percentages, resulting from round 1?

	Strongly disagree	Disagree	Neither nor	Agree	Strongly agree
Approx. 36% of cGvHD patients receive a regimen	❑	❑	❑	❑	❑

containing ruxolitinib, but no ECP.					
Approx. 33% of cGvHD patients receive a regimen containing ECP, but no ruxolitinib.	❑	❑	❑	❑	❑
Approx. 19% of cGvHD patients receive a regimen containing both ECP and ruxolitinib.	❑	❑	❑	❑	❑
Approx. 12% of cGvHD patients receive neither ECP nor ruxolitinib,	❑	❑	❑	❑	❑

Q6.1: Of the steroid refractory aGvHD patients receiving a combination therapy with ECP and ruxolitinib- what percentage start both treatments simultaneously?

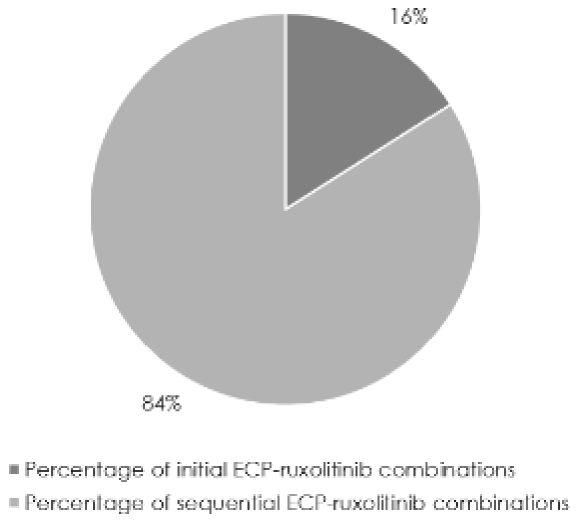

Do you agree on this statement, resulting from round 1?

	Strongly disagree	Disagree	Neither nor	Agree	Strongly agree
Approx. 16% of aGvHD patients receiving a combination therapy start both, ECP and ruxolitinib simultaneously.	❑	❑	❑	❑	❑

Q6.2: Of the steroid refractory cGvHD patients receiving a combination therapy with ECP and ruxolitinib- what percentage start both treatments simultaneously?

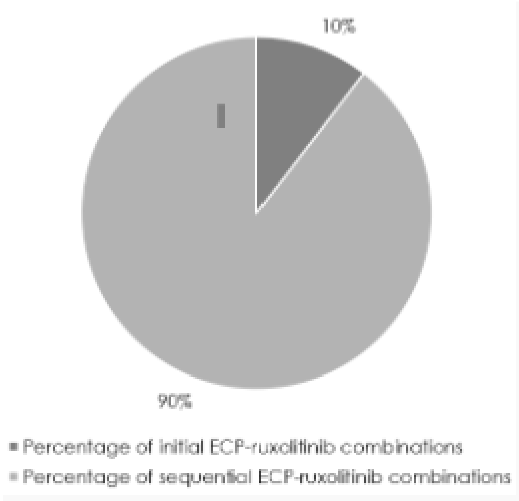

Do you agree on this statement, resulting from the results of round 1?

	Strongly disagree	Disagree	Neither nor	Agree	Strongly agree

Approx. 10% of cGvHD patients receiving a combination therapy start both, ECP and ruxolitinib simultaneously.	❏	❏	❏	❏	❏

Q7.1: What are the main reasons for choosing ECP, but no ruxolitinib, in steroid refractory aGvHD patients? Please rank the reasons resulting from round according to their importance.

- Contraindication and safety profile of ruxolitinib __________
- Ruxolitinib failure __________
- High efficacy of ECP, especially in patients with skin involvement __________
- Safety profile of ECP __________

Q7.2: What are the main reasons for choosing ruxolitinib, but no ECP, in steroid refractory aGvHD patients? Please rank the reasons resulting from round 1 according to their importance.

- High efficacy, especially in patients with gastrointestinal involvement __________
- Regulatory reasons (e.g., EMA approval) __________
- Venous access not necessary __________

Q7.3: What are the main reasons for choosing the combination therapy of ECP and ruxolitinib in steroid refractory aGvHD patients? Please rank the resulting from round 1 according to their importance.

- Increased efficacy __________

- Severe cases __________

Q8.1: What are the main reasons for choosing ECP, but no ruxolitinib, in steroid refractory cGvHD patients? Please rank the reasons resulting from round 1 according to their importance.

- Contraindication to ruxolitinib __________
- Ruxolitinib failure __________
- Efficacy, esp. in patients with skin involvement and for steroid spearing effect __________
- Safety profile __________

Q8.2: What are the main reasons for choosing ruxolitinib, but no ECP, in steroid refractory cGvHD patients? Please rank the reasons resulting from round 1 according to their importance.

- High efficacy, especially in patients with gastrointestinal involvement __________
- Regulatory reasons (e.g., EMA approval) __________
- Patient preferences __________

Q8.3: What are the main reasons for choosing the combination therapy of ECP and ruxolitinib in steroid refractory cGvHD patients? Please rank the reasons resulting from round 1 according to their importance.

- Increased efficacy __________
- Severe cases __________

Q9.1: What is the average time between steroid refractory diagnosis and treatment start in aGvHD for the

treatments below?

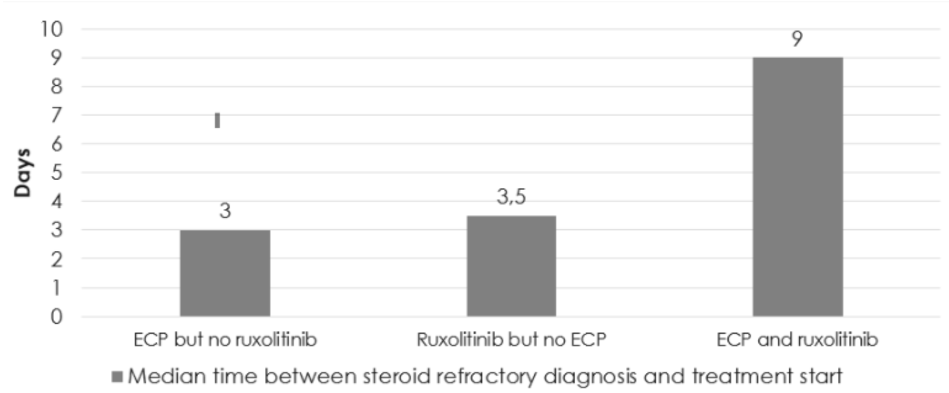

Do you agree on these statements, resulting from round 1?

	Strongly disagree	Disagree	Neither nor	Agree	Strongly agree
In aGvHD patients treated with ECP, the median time between SR diagnosis and treatment start is 3 days.	❑	❑	❑	❑	❑
In aGvHD patients treated with ruxolitinib, the median time between SR diagnosis and treatment start is 3–4 days.	❑	❑	❑	❑	❑
In aGvHD patients treated with both ECP and ruxolitinib, the median time between SR diagnosis and treatment start is 9 days.	❑	❑	❑	❑	❑

Q9.2: What is the average time between steroid refractory diagnosis and treatment start in cGvHD for the treatments below?

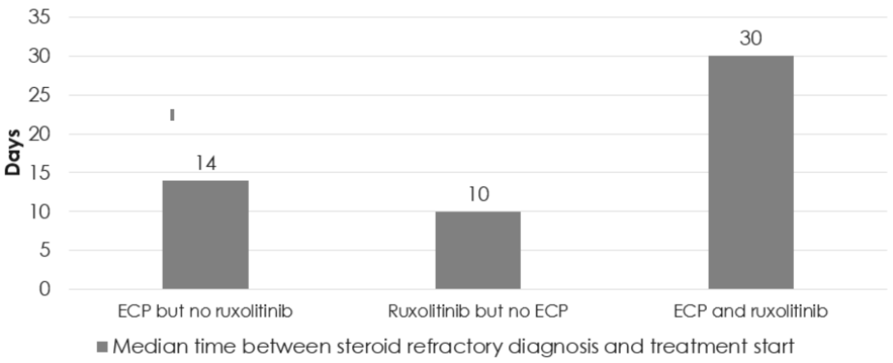

Do you agree on these statements, resulting from round 1?

	Strongly disagree	Disagree	Neither nor	Agree	Strongly agree
In cGvHD patients treated with ECP, the median time between SR diagnosis and treatment start is 14 days.	❑	❑	❑	❑	❑
In cGvHD patients treated with ruxolitinib, the median time between SR diagnosis and treatment start is 10 days.	❑	❑	❑	❑	❑
In cGvHD patients treated with both ECP and ruxolitinib, the median time between SR diagnosis and	❑	❑	❑	❑	❑

treatment start is 30 days.					

Q10.1: Based on your practice and depending on the applied treatment what is the average treatment duration of ECP/ruxolitinib in steroid refractory aGvHD in the following scenarios?

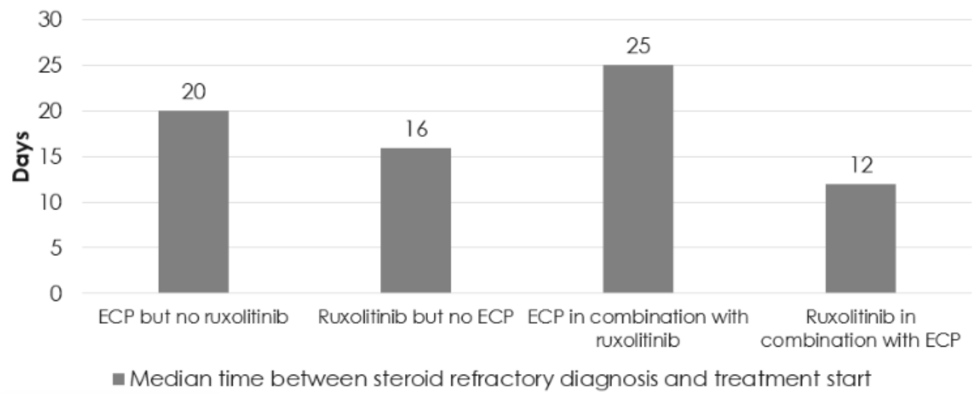

Do you agree on these statements, resulting from round 1?

	Strongly disagree	Disagree	Neither nor	Agree	Strongly agree
The average treatment duration of ECP in aGvHD patients is 20 days.	❑	❑	❑	❑	❑
The average treatment duration of ruxolitinib in aGvHD patients is 16 days.	❑	❑	❑	❑	❑
The average treatment duration of ECP in	❑	❑	❑	❑	❑

combination with ruxolitinib in aGvHD patients is 25 days.					
The average treatment duration of ruxolitinib in combination with ECP in aGvHD patients is 12 days.	❑	❑	❑	❑	❑

Q10.2: Based on your practice and depending on the applied treatment what is the average treatment duration of ECP/ruxolitinib in steroid refractory cGvHD in the following scenarios?

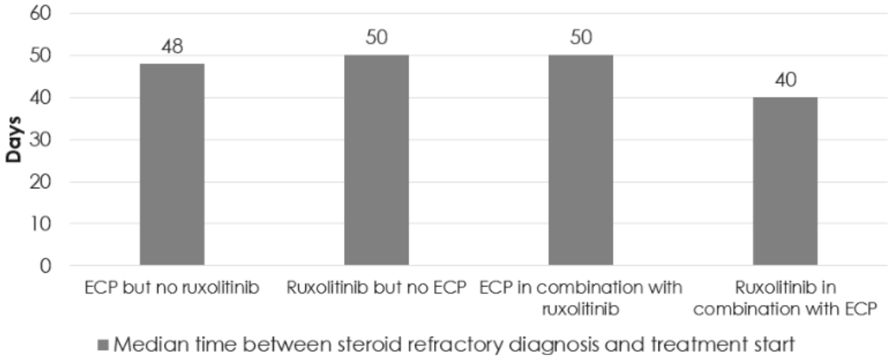

Do you agree on these statements, resulting from round 1?

	Strongly disagree	Disagree	Neither nor	Agree	Strongly agree
The average treatment duration of ECP in cGvHD patients is 48 days.	❑	❑	❑	❑	❑
The average treatment duration of ruxolitinib in cGvHD patients is 50 days.	❑	❑	❑	❑	❑
The average treatment duration of ECP in a combination with ruxolitinib in cGvHD patients is 50 days.	❑	❑	❑	❑	❑

The average treatment duration of ruxolitinib in a combination with ECP in cGvHD patients is 40 days.	❑	❑	❑	❑	❑

Q11.1: How many steroid refractory aGvHD patients switch their treatment?

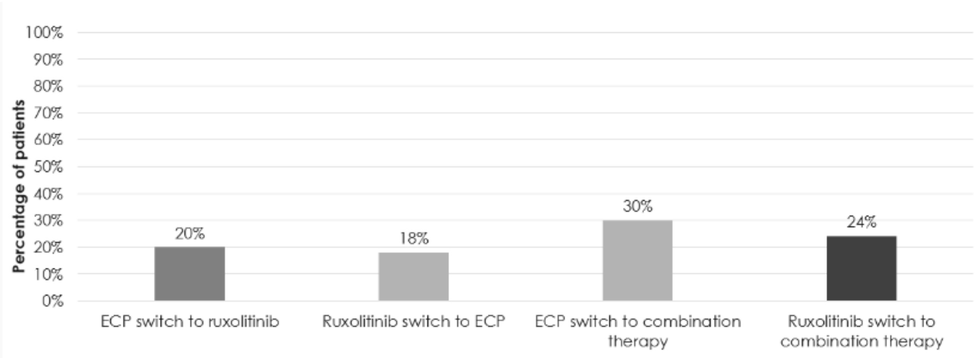

Do you agree on these statements, resulting from round 1?

	Strongly disagree	Disagree	Neither nor	Agree	Strongly agree
On average, 20% of aGvHD patients treated with ECP switch to ruxolitinib.	❑	❑	❑	❑	❑
On average, 18% of aGvHD patients treated with ruxolitinib switch to ECP.	❑	❑	❑	❑	❑
On average, 30% of aGvHD patients treated with ECP switch to a combination	❑	❑	❑	❑	❑

therapy of ECP and ruxolitinib.					
On average, 24% of aGvHD patients treated with ruxolitinib switch to a combination therapy of ruxolitinib and ECP.	❑	❑	❑	❑	❑

Q11.2: How many steroid refractory cGvHD patients switch their treatment?

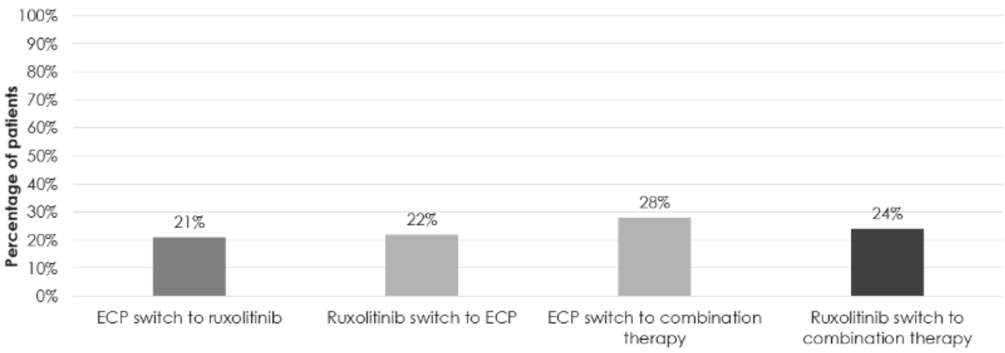

Do you agree on these statements, resulting from round 1?

	Strongly disagree	Disagree	Neither nor	Agree	Strongly agree
On average, 21% of cGvHD patients treated with ECP switch to ruxolitinib.	❑	❑	❑	❑	❑
On average, 22% of cGvHD patients treated with	❑	❑	❑	❑	❑

ruxolitinib switch to ECP.					
On average, 28% of cGvHD patients treated with ECP switch to a combination therapy of ECP and ruxolitinib.	❑	❑	❑	❑	❑
On average, 24% of cGvHD patients treated with ruxolitinib switch to a combination therapy of ruxolitinib and ECP.	❑	❑	❑	❑	❑

Thank you, you have reached the end of the second section.

III. Combination and tapering

Q12.1: Depending on the applied treatment: What is the percentage of steroid refractory aGvHD patients where steroids could be reduced by at least 50%?

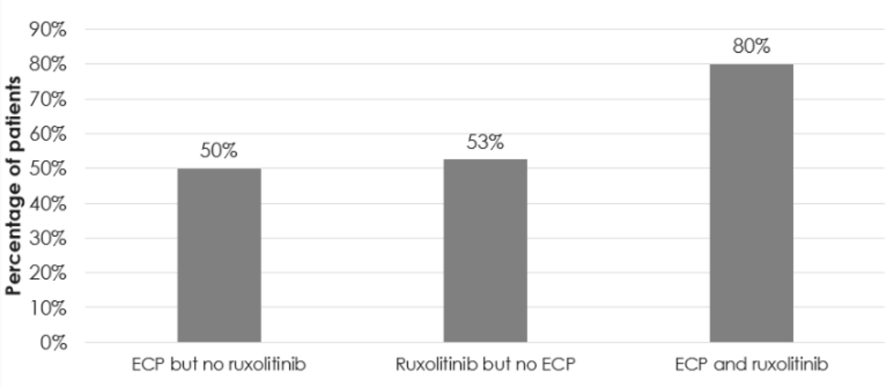

Do you agree on these statements, resulting from round 1?

	Strongly	Disagree	Neither nor	Agree	Strongly

	disagree				agree
In 50% of aGvHD patients treated with ECP, steroids could be reduced by at least 50%.	❑	❑	❑	❑	❑
In 53% of aGvHD patients treated with ruxolitinib, steroids could be reduced by at least 50%.	❑	❑	❑	❑	❑
In 80% of aGvHD patients treated with the combination of ECP and ruxolitinib, steroids could be reduced by at least 50%.	❑	❑	❑	❑	❑

Q12.2: Depending on the applied treatment: What is the percentage of steroid refractory cGvHD patients where steroids could be reduced by at least 50%?

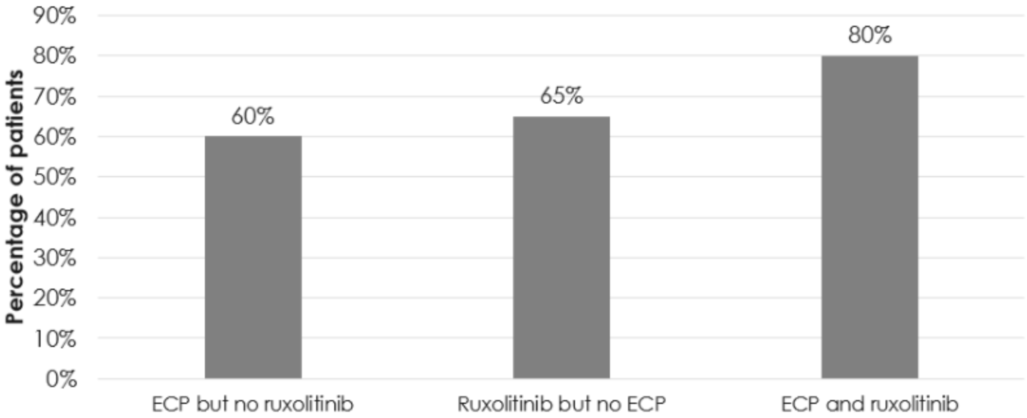

Do you agree on these statements, resulting from round 1?

	Strongly disagree	Disagree	Neither nor	Agree	Strongly agree
In 60% of cGvHD patients treated with ECP, steroids could be reduced by at least 50%.	❑	❑	❑	❑	❑
In 65% of cGvHD patients treated with ruxolitinib, steroids could be reduced by at least 50%.	❑	❑	❑	❑	❑
In 80% of cGvHD patients treated with the combination of ECP and ruxolitinib, steroids could be reduced by at least 50%.	❑	❑	❑	❑	❑

Q13.1: How long would it take before you would be able to implement a 50% reduction of steroid dosage in steroid refractory aGvHD?

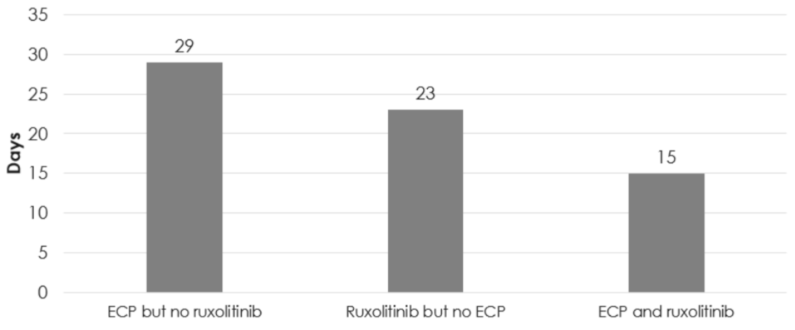

Do you agree on these statements, resulting from round 1?

	Strongly disagree	Disagree	Neither nor	Agree	Strongly agree
On average, it takes 29 days to implement a 50% reduction of steroid dosage in aGvHD patients treated with ECP.	❑	❑	❑	❑	❑
On average, it takes 23 days to implement a 50% reduction of steroid dosage in aGvHD patients treated with ruxolitinib.	❑	❑	❑	❑	❑
On average, it takes 15 days to implement a 50% reduction of steroid dosage in aGvHD patients treated with the combination of ECP and ruxolitinib.	❑	❑	❑	❑	❑

Q13.2: How long would it take before you would be able to implement a 50% reduction of steroid dosage in steroid refractory cGvHD?

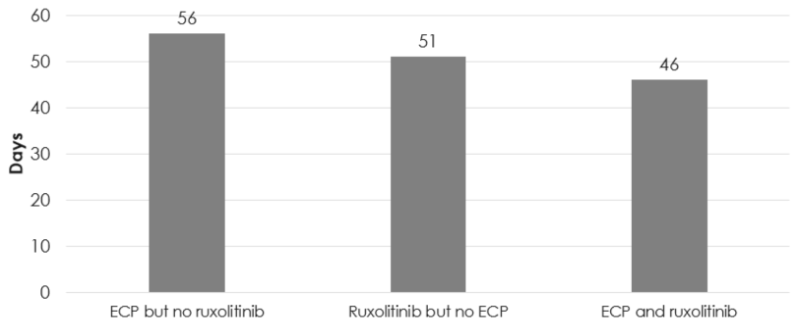

Do you agree on these statements, resulting from round 1?

	Strongly disagree	Disagree	Neither nor	Agree	Strongly agree
On average, it takes 56 days to implement a 50% reduction of steroid dosage in cGvHD patients treated with ECP.	❑	❑	❑	❑	❑
On average, it takes 51 days to implement a 50% reduction of steroid dosage in cGvHD patients treated with ruxolitinib.	❑	❑	❑	❑	❑
On average, it takes 46 days to implement a 50% reduction of steroid dosage in cGvHD patients treated with the combination of ECP and ruxolitinib.	❑	❑	❑	❑	❑

Q14.1: Depending on the applied treatment: What is the percentage of steroid refractory aGvHD patients in your practice where steroid treatment could be stopped completely?

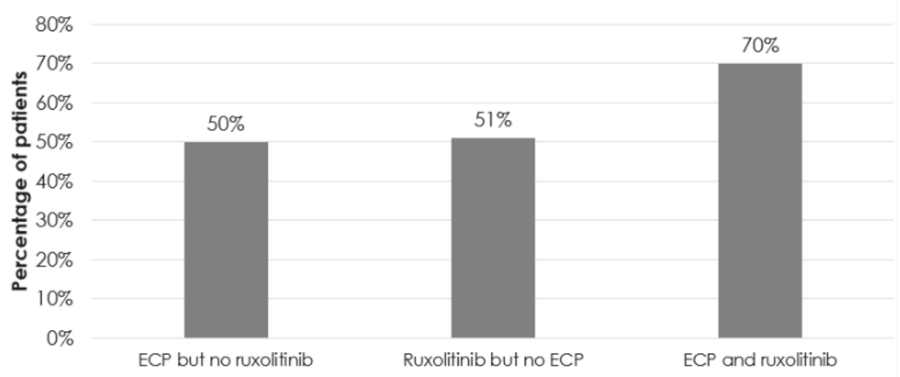

Do you agree on these statements, resulting from round 1?

	Strongly disagree	Disagree	Neither nor	Agree	Strongly agree
On average, steroid treatment could be stopped in 50% of aGvHD patients treated with ECP.	❑	❑	❑	❑	❑
On average, steroid treatment could be stopped in 51% of aGvHD patients treated with ruxolitinib.	❑	❑	❑	❑	❑
On average, steroid treatment could be stopped in 70% of aGvHD	❑	❑	❑	❑	❑

patients treated with the combination of ECP and ruxolitinib.					

Q14.2: Depending on the applied treatment: What is the percentage of steroid refractory cGvHD patients in your practice where steroid treatment could be stopped completely?

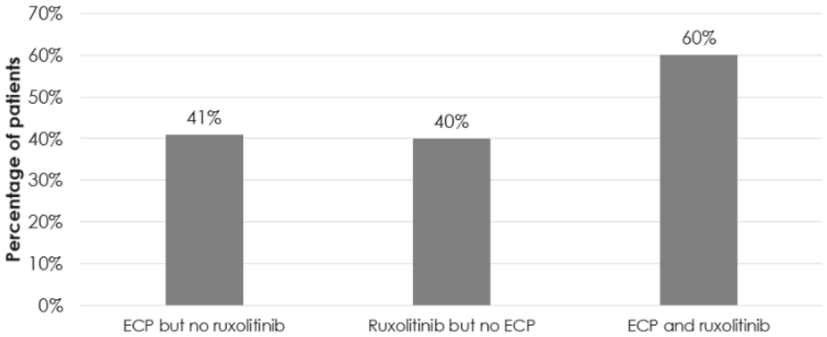

Do you agree on these statements, resulting from round 1?

	Strongly disagree	Disagree	Neither nor	Agree	Strongly agree
On average, steroid treatment could be stopped in 41% of cGvHD patients treated with ECP.	❑	❑	❑	❑	❑
On average, steroid treatment could be stopped in 40% of cGvHD patients treated with	❑	❑	❑	❑	❑

ruxolitinib.					
On average, steroid treatment could be stopped in 60% of cGvHD patients treated with the combination of ECP and ruxolitinib.	❑	❑	❑	❑	❑

Q15.1: How long would it take before you would be able to stop steroid treatment completely in aGvHD?

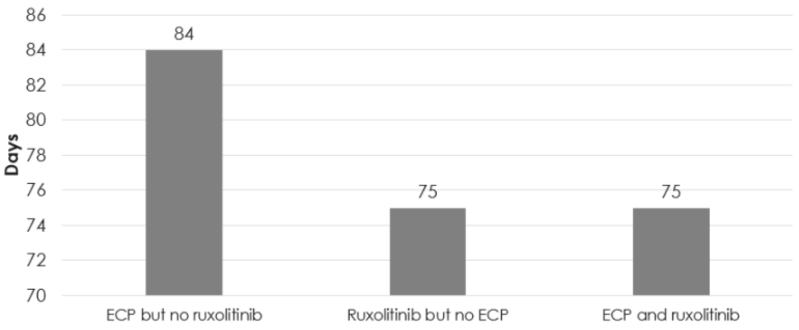

Do you agree on these statements, resulting from round 1?

	Strongly disagree	Disagree	Neither nor	Agree	Strongly agree
On average, it takes 84 days to completely stop steroid treatment in aGvHD patients treated with ECP.	❑	❑	❑	❑	❑
On average, it takes 75 to completely stop steroid	❑	❑	❑	❑	❑

treatment in aGvHD patients treated with ruxolitinib.					
On average, it takes 75 days to completely stop steroid treatment in aGvHD patients treated with the combination of ECP and ruxolitinib.	❑	❑	❑	❑	❑

Q15.2: How long would it take before you would be able to stop steroid treatment completely in cGvHD?

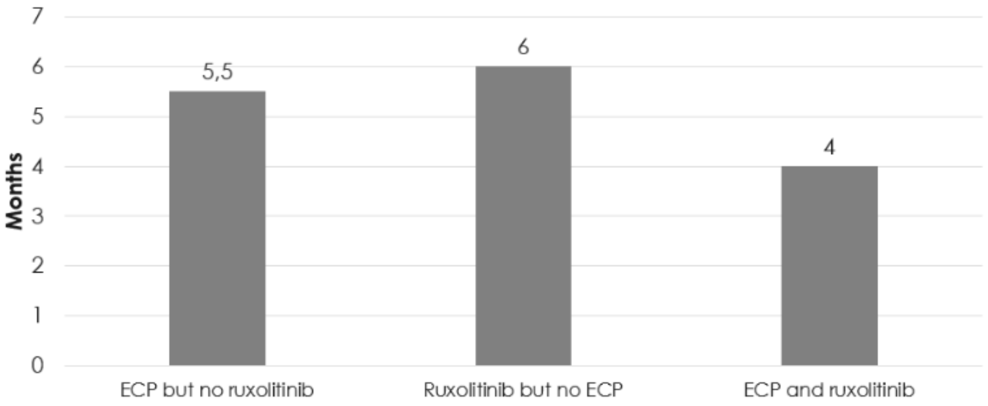

Do you agree on these statements, resulting from round 1?

	Strongly disagree	Disagree	Neither nor	Agree	Strongly agree
On average, it takes 5.5 months to completely stop steroid treatment in cGvHD	❑	❑	❑	❑	❑

patients treated with ECP.					
On average, it takes 6 months to completely stop steroid treatment in cGvHD patients treated with ruxolitinib.	❑	❑	❑	❑	❑
On average, it takes 4 months to completely stop steroid treatment in cGvHD patients treated with the combination of ECP and ruxolitinib.	❑	❑	❑	❑	❑

Q16.1: In the following, two sets of criteria for the definition of ruxolitinib refractoriness in aGvHD patients are presented. Please choose which of these sets you fits your current practice and you align with.

Set 1 – Mohty et al. (Mohty M, Holler E, Jagasia M, Jenq R, Malard F, Martin P, Socié G, Zeiser R. Refractory acute graft-versus-host disease: a new working definition beyond corticosteroid refractoriness. Blood. 2020 Oct 22;136(17):1903-1906):

- Progression of GvHD compared to baseline after at least 5 to 10 days of treatment with ruxolitinib, based either on objective increase in stage/grade, or new organ involvement;
- Lack of improvement in GvHD (PR or better) compared to baseline after at least 14 days of treatment with ruxolitinib; or

- Loss of response, defined as objective worsening of GvHD determined by increase in stage, grade or new organ involvement at any time after initial improvement.

Set 2 – Modified criteria based on answers from round 1:
- Progression of GvHD compared to baseline in any organ after at least 3 to 5 days of treatment with ruxolitinib, based either on objective increase in stage/grade, or new organ involvement;
- Lack of improvement in GvHD (PR or better) compared to baseline within 5-7 days of treatment with ruxolitinib;
- Loss of response, defined as objective worsening of GvHD determined by increase in stage, grade or new organ involvement at any time after initial improvement.

	Strongly disagree	Disagree	Neither nor	Agree	Strongly agree
Set 1 – Mohty et al.	❏	❏	❏	❏	❏
Set 2 – Modified criteria based on answers from round one	❏	❏	❏	❏	❏

You disagreed on both criteria. Please enter your definition of ruxolitinib refractoriness in aGvHD patients that fits your current practice and you align with.

Q16.2: How do you determine that a cGvHD patient is refractory to ruxolitinib? Do you agree on these criteria, resulting from round 1?

	Strongly disagree	Disagree	Neither nor	Agree	Strongly agree
Progression of GvHD compared to baseline after 1-2 weeks of treatment with ruxolitinib, based either on objective increase in stage/grade, or new organ involvement	❑	❑	❑	❑	❑
Lack of improvement in GvHD (PR or better) compared to baseline after 2-3 month of treatment with ruxolitinib	❑	❑	❑	❑	❑
Loss of response, defined as objective worsening of GvHD determined by	❑	❑	❑	❑	❑

increase in severity or new organ involvement at any time after initial improvement.					

You disagreed on the criteria from round 1. How do you determine that a cGvHD patient is refractory to ruxolitinib?

Q17.1: What is the average treatment time on ruxolitinib before ruxolitinib refractoriness in aGvHD is established? Do you agree on this statement, resulting from round 1?

	Strongly disagree	Disagree	Neither nor	Agree	Strongly agree
It takes aGvHD patients, on average, 14 days on ruxolitinib before ruxolitinib refractoriness is established.	❑	❑	❑	❑	❑

Q17.2: What is the average treatment time on ruxolitinib before

ruxolitinib refractoriness in cGvHD is established? Do you agree on this statement, resulting from round 1?

	Strongly disagree	Disagree	Neither nor	Agree	Strongly agree
It takes cGvHD patients, on average, 60 days on ruxolitinib before ruxolitinib refractoriness is established.	❑	❑	❑	❑	❑

Q18.1: When treating steroid refractory aGvHD patients with ECP, what would be reasons for stopping ECP treatment? Do you agree on the ranking below, resulting from round 1?

	Strongly disagree	Disagree	Neither nor	Agree	Strongly agree
Rank 1 (most important): Lack of efficacy	❑	❑	❑	❑	❑
Rank 2: Inconvenience for the patient	❑	❑	❑	❑	❑

Rank 3 (least important): Side effects	❑	❑	❑	❑	❑

You disagreed on the ranking from round 1. When treating steroid refractory aGvHD patients with ECP, what would be reasons for stopping ECP treatment?

- Inconvenience for the patient __________
- Lack of efficacy __________
- Side effects __________
- Another reason, namely: __________

Q18.2: When treating steroid refractory cGvHD patients with ECP, what would be reasons for stopping ECP treatment? Do you agree on the ranking below, resulting from round 1?

	Strongly disagree	Disagree	Neither nor	Agree	Strongly agree
Rank 1 (most important): Lack of efficacy	❑	❑	❑	❑	❑
Rank 2: Inconvenience for the patient	❑	❑	❑	❑	❑
Rank 3 (least important): Other reasons, e.g., loss of venous access or failure to reduce steroid dosage	❑	❑	❑	❑	❑

You disagreed on the ranking from round 1. When treating steroid refractory cGvHD patients with ECP, what would be reasons for stopping ECP treatment?

- Inconvenience for the patient __________
- Lack of efficacy __________
- Side effects __________
- Another reason, namely: __________

Q19.1: When treating steroid refractory aGvHD patients with ruxolitinib, what would be reasons for stopping ruxolitinib treatment? Do you agree on the ranking below, resulting from round 1?

	Strongly disagree	Disagree	Neither nor	Agree	Strongly agree
Rank 1: Lack of efficacy	❑	❑	❑	❑	❑
Rank 2: Side effects	❑	❑	❑	❑	❑
Rank 3: Other reasons, e.g., failure to stop or reduce steroid dosage	❑	❑	❑	❑	❑

You disagreed with the ranking from round 1. When treating steroid refractory aGvHD patients with ruxolitinib, what would be reasons for stopping ruxolitinib treatment?

- Inconvenience for the patient __________
- Lack of efficacy __________
- Side effects __________
- Another reason, namely: __________

Q19.2: When treating steroid refractory cGvHD patients with ruxolitinib, what would be reasons for stopping ruxolitinib treatment? Do you agree on the ranking below, resulting from round 1?

	Strongly disagree	Disagree	Neither nor	Agree	Strongly agree
Rank 1: Lack of efficacy	❑	❑	❑	❑	❑
Rank 2: Side effects	❑	❑	❑	❑	❑
Rank 3: Other reasons, e.g., failure to reduce steroid dosage	❑	❑	❑	❑	❑

You disagreed on the ranking from round 1. When treating steroid refractory cGvHD patients with ruxolitinib, what would

be the reasons for stopping ruxolitinib treatment?

- Inconvenience for the patient __________
- Lack of efficacy __________
- Side effects __________
- Another reason, namely: __________

Q20.1: When treating steroid refractory aGvHD patients with combination therapy (ECP + ruxolitinib), what would be reasons to stop the combination treatment? Do you agree on the ranking below, resulting from round 1?

	Strongly disagree	Disagree	Neither nor	Agree	Strongly agree
Rank 1: Lack of efficacy	❑	❑	❑	❑	❑
Rank 2: Side effects	❑	❑	❑	❑	❑
Rank 3: Other reasons, e.g., failure to reduce steroid dosage	❑	❑	❑	❑	❑

You disagreed on the ranking from round 1. When treating steroid refractory aGvHD patients with combination therapy (ECP & ruxolitinib), what would be reasons to stop the combination treatment?

- Inconvenience for the patient __________
- Lack of efficacy __________
- Side effects __________
- Another reason, namely: __________

Q20.2: When treating steroid refractory cGvHD patients with combination therapy (ECP + ruxolitinib), what would be reasons to stop the combination treatment? Do you agree on the ranking below, resulting from round 1?

	Strongly disagree	Disagree	Neither nor	Agree	Strongly agree
Rank 1: Lack of efficacy	❑	❑	❑	❑	❑
Rank 2: Side effects	❑	❑	❑	❑	❑
Rank 3: Other reasons, e.g., failure to reduce steroid dosage	❑	❑	❑	❑	❑

You disagreed on the ranking from round 1. When treating steroid refractory cGvHD patients with combination treatment (ECP & ruxolitinib), what would be reasons to stop the combination treatment?

- Inconvenience for the patient __________
- Lack of efficacy __________

- Side effects __________
- Another reason, namely: __________

Q21.1: Please list the reasons you would NOT add ECP to ruxolitinib in steroid refractory aGvHD patients. Do you agree on the ranking below, resulting from round 1?

	Strongly disagree	Disagree	Neither nor	Agree	Strongly agree
Rank 1: Inconvenience for the patient	❑	❑	❑	❑	❑
Rank 2: Other reasons, e.g., lack of venous access or low white blood cell count	❑	❑	❑	❑	❑
Rank 3: Low efficacy	❑	❑	❑	❑	❑

You disagreed on the ranking from round 1. Please list the reasons you would NOT add ECP to ruxolitinib in steroid refractory aGvHD patients. Please order the reasons from most important to least important.

- Low efficacy __________
- Inconvenience for the patient __________
- High workload for the hospital staff __________
- ECP capacity in my centre is restricted __________
- Other reason, please specify __________

Q21.2: Please list the reasons you would NOT add ECP to ruxolitinib in steroid refractory cGvHD patients. Do you agree on the ranking below, resulting from round 1?

	Strongly disagree	Disagree	Neither nor	Agree	Strongly agree
Rank 1: Inconvenience for the patient	❑	❑	❑	❑	❑
Rank 2: High workload for the hospital staff	❑	❑	❑	❑	❑
Rank 3: Restricted ECP capacity	❑	❑	❑	❑	❑

You disagreed on the ranking from round 1. Please list the reasons you would NOT add ECP to ruxolitinib in steroid refractory cGvHD patients. Please order the reasons from most important to least important.

- Low efficacy __________
- Inconvenience for the patient __________
- High workload for the hospital staff __________
- ECP capacity in my centre is restricted __________
- Other reason, please specify __________

Q22.1: What is the average time period until addition of ruxolitinib or ECP in aGvHD?

Do you agree on these statements, resulting from round 1?

	Strongly disagree	Disagree	Neither nor	Agree	Strongly agree
The average time period from ECP to addition of ruxolitinib is 14 days in aGvHD patients.	❑	❑	❑	❑	❑
The average time period from ruxolitinib to addition of ECP is 14 days in aGvHD patients.	❑	❑	❑	❑	❑

Q22.2: What is the average time period until addition of ruxolitinib or ECP is cGvHD?

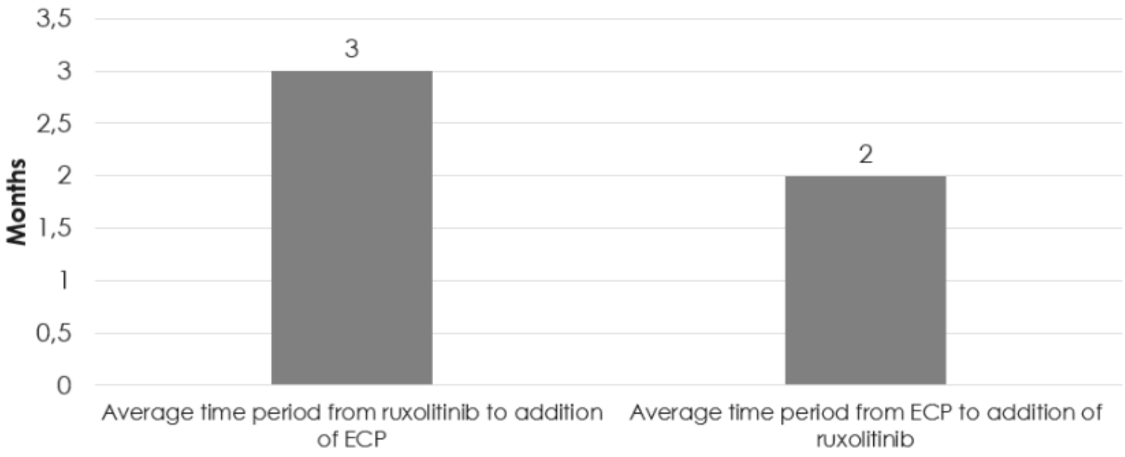

Do you agree on these statements, resulting from round 1?

	Strongly disagree	Disagree	Neither nor	Agree	Strongly agree
The average time period from ECP to addition of ruxolitinib is 3 months in cGvHD patients.	❑	❑	❑	❑	❑
The average time period from ruxolitinib to addition of ECP is 2 months in cGvHD patients.	❑	❑	❑	❑	❑

Q23.1: Please list the advantages/ of using a sequential treatment regimen of ECP and ruxolitinib in aGvHD.Do you agree on this ranking, resulting from round 1?

	Strongly disagree	Disagree	Neither nor	Agree	Strongly agree
Rank 1: Improved response (incl. faster and more durable response)	❑	❑	❑	❑	❑
Rank 2: Safety aspects (e.g., less side effext, fewer hospitalization, no increase in toxicity)	❑	❑	❑	❑	❑

Rank 3: Steroid sparing effect (incl. faster steroid tapering)	❑	❑	❑	❑	❑

According to your opinion: Please list the advantages/ of using a sequential treatment regimen of ECP and ruxolitinib in aGvHD.

Q23.2: Please list the advantages of using a sequential treatment regimen of ECP and ruxolitinib in cGvHD. Do you agree on this ranking, resulting from round 1?

	Strongly disagree	Disagree	Neither nor	Agree	Strongly agree
Rank 1: Improved response (incl. faster and more durable response)	❑	❑	❑	❑	❑
Rank 2: Safety aspects (e.g., less side effects, fewer hospitalization, no increase	❑	❑	❑	❑	❑

in toxicity)					
Rank 3: Steroid sparing effects (incl. faster steroid tapering)	❑	❑	❑	❑	❑

You disagreed on the ranking from round 1. Please list and rank the advantages of using a sequential treatment regimen of ECP and ruxolitinib in cGvHD.

- Improved response (incl. faster and more durable response) __________
- Safety aspects (e.g., less side effects, fewer hospitalization, no increase in toxicity) __________
- Steroid sparing effects (incl. faster steroid tapering) __________
- Other, namely: __________

Q24.1: If steroid refractory aGvHD patients respond to the combination therapy of ECP and ruxolitinib you may want to reduce treatment. How would you design the reduction of combination therapy?

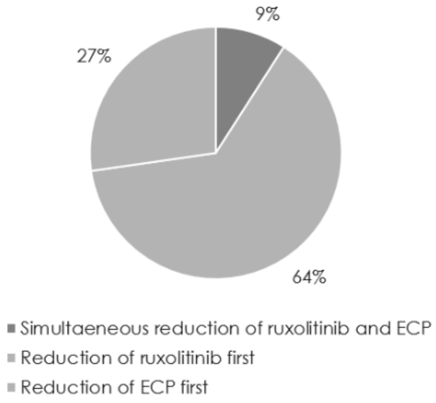

Do you agree on these statements resulting, from round 1?

	Strongly disagree	Disagree	Neither nor	Agree	Strongly agree
When reducing combination therapy in aGvHD patients, ruxolitinib is reduced first in 64% of cases.	❑	❑	❑	❑	❑
When reducing combination therapy in aGvHD patients, ECP is reduced first in 27% of cases.	❑	❑	❑	❑	❑
When reducing combination therapy in aGvHD patients, ruxolitinib and ECP are reduced simultaneously in 9% of cases.	❑	❑	❑	❑	❑

Q24.2: If steroid refractory cGvHD patients respond to the combination therapy of ECP and ruxolitinib you may want to reduce treatment. How would you design the reduction of combination therapy?

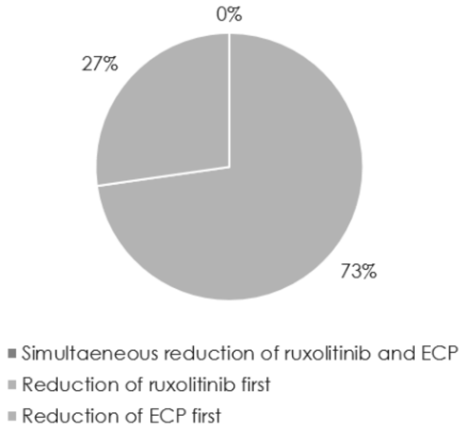

Do you agree on these statements, resulting from round 1?

	Strongly disagree	Disagree	Neither nor	Agree	Strongly agree
When reducing combination therapy in cGvHD patients, ruxolitinib is reduced first in 73% of cases.	❑	❑	❑	❑	❑
When reducing combination therapy in cGvHD patients, ECP is reduced first in 27% of cases.	❑	❑	❑	❑	❑
When reducing combination therapy in cGvHD, there is no simultaneously reduction of ruxolitinib and ECP.	❑	❑	❑	❑	❑

Q25.1: List the 3 therapies you are most likely to combine with ECP in aGvHD. Do you agree on this ranking, resulting from round 1?

	Strongly disagree	Disagree	Neither nor	Agree	Strongly agree
Rank 1 (most likely): Ruxolitinib	❑	❑	❑	❑	❑
Rank 2: Fecal microbiota transplantation	❑	❑	❑	❑	❑
Rank 3: TNF-alpha inhibitors	❑	❑	❑	❑	❑
Rank 4: mTor inhibitors, ATG	❑	❑	❑	❑	❑
Rank 5 (Least likely): Others, e.g. Ibrutinib, Vedolizumab, alpha-1-antitrypsin, etanercept	❑	❑	❑	❑	❑

You disagreed on the ranking from round 1. Please list and rank the 3 therapies you are most likely to combine with ECP in aGvHD.

- Ruxolitinib __________
- Fecal microbiota transplantation __________
- TNF-alpha inhibitors __________

- mTor inhibitors, ATG 4 __________
- Others, e.g. Ibrutinib, Vedolizumab, alpha-1-antitrypsin, etanercept __________

Q25.2: List the 3 therapies you are most likely to combine with ECP in cGvHD. Do you agree on this ranking, resulting from round 1?

	Strongly disagree	Disagree	Neither nor	Agree	Strongly agree
Rank 1 (most likely): Ruxolitinib	❑	❑	❑	❑	❑
Rank 2: mTOR inhibitors	❑	❑	❑	❑	❑
Rank 3 (least likely): Ibrutinib, belumosudil, TNF-alpha inhibitors, mycophenolate mofetil	❑	❑	❑	❑	❑

You disagreed on the ranking from round 1. Please list and rank the 3 therapies you are most likely to combine with ECP in cGvHD.

- Ruxolitinib __________
- mTOR inhibitors __________
- Ibrutinib, belumosudil, TNF-alpha inhibitors, mycophenolate mofetil __________

- Others, namely __________

Q26: What would be the hurdles preventing a timely use of ECP after steroid refractoriness has been established? Do you agree on this ranking, resulting from round 1?

	Strongly disagree	Disagree	Neither nor	Agree	Strongly agree
Rank 1 (most important): Difficulties with venous access for ECP	❑	❑	❑	❑	❑
Rank 2: Restricted capacity of ECP unit	❑	❑	❑	❑	❑
Rank 3 (least important): Patient decisions	❑	❑	❑	❑	❑

You disagreed on the ranking from round 1. What would be the hurdles preventing a timely use of ECP after steroid refractoriness has been established? Please rank the following reasons from most important to least important.

- Restricted capacity of the ECP unit __________
- Restricted hospital bed capacity __________
- Difficulties with venous access for ECP __________
- Patient decision __________

- Different reason/hurdle: (please indicate) __________

Thank you, you have reached the end of the third section.

IV. Exploratory questions on ECP

Q27: Do you see the potential of using ECP as a monotherapy treatment (without steroids) in aGvHD patients?*

*Please note: For this question you can also consider low grade aGvHD patients.

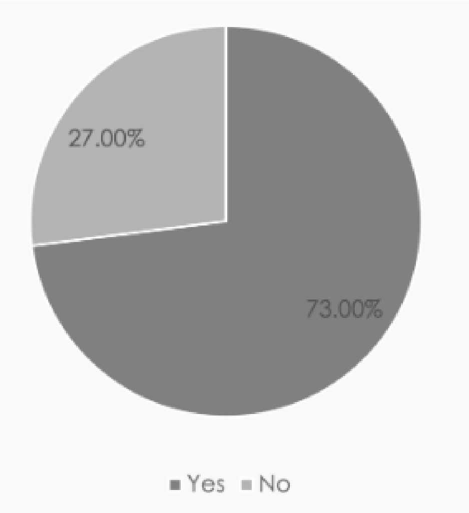

Do you agree on this statement, resulting from round 1?

	Strongly disagree	Disagree	Neither nor	Agree	Strongly agree
ECP shows the potential to be used as a monotherapy (without steroids) in aGvHD patients.	❑	❑	❑	❑	❑

Q28.1: In the first round of questioning, you indicated that you

see the potential for ECP monotherapy (without steroids) in cGvHD patients (91% consensus among participants). Please list the selection criteria to treat aGvHD patients with ECP monotherapy.

Q28.2: In the first round of questioning, you also indicated that you see the potential for ECP monotherapy (without steroids) in cGvHD patients. Please list the selection criteria to treat cGvHD patients with ECP monotherapy.

Q29.1: What percentage of aGvHD patients would you consider treating with ECP even if they are not refractory to steroids? Do you agree on this statement, resulting from round 1?

	Strongl<br>y	Disagr<br>ee	Neithe<br>r nor	Agree	Strongl<br>y

	disagree				agree
In 89pprox.. 25% of aGvHD patients, ECP is considered even if they are not steroid refractory.	❑	❑	❑	❑	❑

Q29.2: What percentage of cGvHD patients would you consider treating with ECP even if they are not refractory to steroids? Do you agree on this statement, resulting from round 1?

	Strongly disagree	Disagree	Neither nor	Agree	Strongly agree
In 89pprox.. 27% of cGvHD patients, ECP is considered even if they are not steroid refractory.	❑	❑	❑	❑	❑

Delphi study round 3 questionnaire

Dear participant,

welcome to the third round of the Delphi Panel on the use of extracorporeal photopheresis (ECP) in steroid-refractory acute graft-versus-host disease (aGvHD) in grades II-IV and moderate-severe chronic graft-versus-host disease (cGvHD) following HSCT.

We would like to thank you for responding to the first and second questionnaires.

We evaluated and summarized the results of the first two rounds to develop this new version. The aim of this third round of questioning is to vote on the results of the first/second round and ideally to reach a consensus.

Please allow sufficient time to complete the questionnaire. The estimated time required is 60-120 minutes. Your participation in this study will remain anonymous to the other participants.

We would like to thank you in advance for your participation and support!

Your study team

Q1.1: When treating steroid-refractory aGvHD patients with ECP alone* but not with ruxolitinib, participants agreed in the second round that they initially treat patients with 2-3 ECP procedures per week on consecutive days weekly for 4

weeks. Which treatment schedules do you apply afterwards?* +/- steroids and/or CNIDo you agree on these statements, resulting from round 1 and 2?

	Strongly disagree	Disagree	Neither nor	Agree	Strongly agree
Treatment schedule 2: 2 ECP procedures per week at least every two weeks for approximately 8 weeks (2 months)	❑	❑	❑	❑	❑
Treatment schedule 3: 2 ECP procedures per week at least every month for approximately 8 weeks (2 months)	❑	❑	❑	❑	❑

You disagreed on the statement. When treating steroid-refractory aGvHD patients with ECP alone* - which treatment schedules do you apply?*+/- steroids and/or CNI

	Treatment

	schedule
Treatment schedule 2	❑
Treatment schedule 3	❑

Q1.2 When treating steroid-refractory cGvHD patients with ECP alone* - which treatment schedules do you apply?*+/- steroids and/or CNIDo you agree on these statements, resulting from round 1 and 2?

	Strongly disagree	Disagree	Neither nor	Agree	Strongly agree
Treatment schedule 1: 2 ECP procedures per week for approximately 9 weeks	❑	❑	❑	❑	❑
Treatment schedule 2: 2 ECP procedures per week , at least every two weeks for approximately 10 weeks	❑	❑	❑	❑	❑
Treatment schedule 3: 1-2 ECP procedures per week	❑	❑	❑	❑	❑

at least monthly for approximately 20 weeks (5 months)					

You disagreed on the statement. When treating steroid-refractory cGvHD patients with ECP alone* - which treatment schedules (1-3) do you apply?*+/- steroids and/or CNI

	Treatment schedule
Treatment schedule 1	❑
Treatment schedule 2	❑
Treatment schedule 3	❑

Q2: What is the percentage of patients with steroid-refractory cGvHD that receive various treatments as per described below? Do you agree on the percentages, resulting from round 2?

	Strongly disagree	Disagree	Neither nor	Agree	Strongly agree
Approx. 30% – 40% of cGvHD patients receive a regimen containing ruxolitinib, but no ECP.	❑	❑	❑	❑	❑
Approx. 20% – 30% of cGvHD patients receive a regimen containing ECP, but no ruxolitinib.	❑	❑	❑	❑	❑
Approx. 10% – 20% of cGvHD patients receive a regimen containing both ECP and ruxolitinib.	❑	❑	❑	❑	❑
Approx. 10% – 20% of cGvHD patients receive neither ECP nor ruxolitinib,	❑	❑	❑	❑	❑

Q3.1: What are the main reasons for choosing ECP, but no ruxolitinib, in steroid-refractory aGvHD patients? Do you agree on the ranking, resulting from round 2?

	Strongly disagree	Disagree	Neither nor	Agree	Strongly agree
Rank 1: Contraindication and safety profile of ruxolitinib	❑	❑	❑	❑	❑
Rank 2: Ruxolitinib failure	❑	❑	❑	❑	❑
Rank 3: Safety profile of ECP and high efficacy of ECP (especially in patients with skin involvement)	❑	❑	❑	❑	❑

You disagreed on the ranking from round 2. What are the main reasons for choosing ECP, but no ruxolitinib, in steroid-refractory aGvHD patients?

- Contraindication and safety profile of ruxolitinib __________
- Ruxolitinib failure __________
- High efficacy of ECP, especially in patients with skin involvement __________
- Safety profile of ECP __________
- Other __________

Q3.2: What are the main reasons for choosing ruxolitinib, but no ECP, in steroid-refractory aGvHD patients? Do you agree on the following ranking from round 2?

	Strongly disagree	Disagree	Neither nor	Agree	Strongly agree
Rank 1: High efficacy, especially in patients with gastrointestinal involvement	❑	❑	❑	❑	❑
Rank 2: Regulatory reasons (e.g., EMA approval)	❑	❑	❑	❑	❑
Rank 3: Venous access not necessary	❑	❑	❑	❑	❑

You disagreed on the ranking from round 2. What are the main reasons for choosing ruxolitinib, but no ECP, in steroid-refractory aGvHD patients?

- High efficacy, especially in patients with gastrointestinal involvement __________
- Regulatory reasons (e.g., EMA approval) __________
- Venous access not necessary __________

- Other __________

Q3.3: What are the main reasons for choosing the combination therapy of ECP and ruxolitinib in steroid-refractory aGvHD patients?Do you agree on the ranking from round 2?

	Strongly disagree	Disagree	Neither nor	Agree	Strongly agree
Rank 1: Severe cases	❑	❑	❑	❑	❑
Rank 2: Increased efficacy	❑	❑	❑	❑	❑

You disagreed on the ranking from round 2. What are the main reasons for choosing the combination therapy of ECP and ruxolitinib in steroid-refractory aGvHD patients?

- Increased efficacy __________
- Severe cases __________
- Other __________

Q4.1: What are the main reasons for choosing ECP, but no ruxolitinib, in steroid-refractory cGvHD patients?Do you agree with the ranking from round 2?

	Strongly disagree	Disagree	Neither nor	Agree	Strongly agree
Rank 1: Contraindication to ruxolitinib	❑	❑	❑	❑	❑
Rank 2: Ruxolitinib failure	❑	❑	❑	❑	❑
Rank 3: Efficacy, esp. in patients with skin involvement and for steroid sparing effect	❑	❑	❑	❑	❑
Rank 4: Safety profile	❑	❑	❑	❑	❑

You disagreed with the ranking from round 2. What are the main reasons for choosing ECP, but no ruxolitinib, in steroid-refractory cGvHD patients?

- Contraindication to ruxolitinib __________
- Ruxolitinib failure __________

- Efficacy, esp. in patients with skin involvement and for steroid sparing effect __________
- Safety profile __________
- Other __________

Q4.2: What are the main reasons for choosing ruxolitinib, but no ECP, in steroid-refractory cGvHD patients?Do you agree with the ranking from round 2?

	Strongly disagree	Disagree	Neither nor	Agree	Strongly agree
Rank 1: High efficacy, especially in patients with gastrointestinal involvement	❑	❑	❑	❑	❑
Rank 2: Regulatory reasons (e.g., EMA approval)	❑	❑	❑	❑	❑
Rank 3: Patient preferences	❑	❑	❑	❑	❑

You disagreed with the ranking from round 2. What are the main reasons for choosing ruxolitinib, but no ECP, in steroid-

refractory cGvHD patients?

- High efficacy, especially in patients with gastrointestinal involvement __________
- Regulatory reasons (e.g., EMA approval) __________
- Patient preferences __________
- Other __________

Q4.3: What are the main reasons for choosing the combination therapy of ECP and ruxolitinib in steroid-refractory cGvHD patients?Do you agree with the ranking from round 2?

	Strongly disagree	Disagree	Neither nor	Agree	Strongly agree
Rank 1: Increased efficacy	❑	❑	❑	❑	❑
Rank 2: Severe cases	❑	❑	❑	❑	❑

You disagreed with the ranking from round 2. What are the main reasons for choosing the combination therapy of ECP and ruxolitinib in steroid-refractory cGvHD patients?

- Increased efficacy __________
- Severe cases __________
- Other __________

Q5: What is the average time between diagnosis of steroid-refractory aGvHD and the treatment start of ruxolitinib?Do you agree on the statement, resulting from round 2?

	Strongly disagree	Disagree	Neither nor	Agree	Strongly agree
In aGvHD patients treated with ruxolitinib, the time between SR diagnosis and treatment start is maximum 4 days.	❑	❑	❑	❑	❑

Q6.1: Based on your practice and depending on the applied treatment, what is the average treatment duration of ECP/ruxolitinib in steroid-refractory aGvHD in the following scenarios?Do you agree on these statements, resulting from round 1 and round 2?

	Strongly disagree	Disagree	Neither nor	Agree	Strongly agree
The average treatment duration of ECP is approximately 4-6 months.	❑	❑	❑	❑	❑
The average treatment duration of ruxolitinib is approximately 3-5 months.	❑	❑	❑	❑	❑
The average treatment duration of ECP in combination with ruxolitinib is approximately 4-6 months.	❑	❑	❑	❑	❑
The average treatment duration of ruxolitinib in combination with ECP is approximately 3-5 months.	❑	❑	❑	❑	❑

Q6.2: Based on your practice and depending on the applied treatment, what is the average treatment duration of ECP/ruxolitinib in steroid-refractory cGvHD in the following scenarios?Do you agree on these statements,

resulting from round 1 and round 2?

	Strongly disagree	Disagree	Neither nor	Agree	Strongly agree
The average treatment duration of ECP is approximately 10-12 months.	❑	❑	❑	❑	❑
The average treatment duration of ruxolitinib is approximately 10-12 months.	❑	❑	❑	❑	❑
The average treatment duration of ECP in combination with ruxolitinib is approximately 8-10 months.	❑	❑	❑	❑	❑
The average treatment duration of ruxolitinib in combination with ECP is approximately 8-10 months.	❑	❑	❑	❑	❑

Q7: How long would it take before you would be able to stop

steroid treatment completely in aGvHD?Do you agree on these statements, resulting from round 2?

	Strongly disagree	Disagree	Neither nor	Agree	Strongly agree
On average, it takes 2–3 months to completely stop steroid treatment in patients treated with ECP.	❑	❑	❑	❑	❑
On average, it takes 2–3 months to completely stop steroid treatment in patients treated with ruxolitinib.	❑	❑	❑	❑	❑
On average, it takes 2–3months to completely stop steroid treatment in patients treated with the combination of ECP and ruxolitinib.	❑	❑	❑	❑	❑

Q8: Would you agree on the following definition of ruxolitinib refractoriness in aGvHD: Progression of GvHD compared to

baseline in any organ after at least 5 days of treatment with ruxolitinib, based either on objective increase in stage/grade, or new organ involvement;Lack of improvement in GvHD (PR or better) compared to baseline within 5-10 days of treatment with ruxolitinib;Loss of response, defined as objective worsening of GvHD determined by increase in stage, grade or new organ involvement at any time after initial improvement.

	Strongly disagree	Disagree	Neither nor	Agree	Strongly agree
The definition above defines ruxolitinib refractoriness in acute GvHD.	❑	❑	❑	❑	❑

Q9: Please list the reasons you would NOT add ECP to ruxolitinib in steroid-refractory cGvHD patients.Do you agree on the ranking below, resulting from round 2?

	Strongly disagr	Disagree	Neither nor	Agree	Strongly agree

	ee				
Rank 1: Inconvenience for the patient	❑	❑	❑	❑	❑
Rank 2: Organizational/logistic reasons (such as restricted hospital capacity)	❑	❑	❑	❑	❑

Q10.3: In round 1 and 2, you agreed that ruxolitinib is the treatment that is most likely combined with ECP in acute GvHD. Do you have any objections combining ECP with any other guideline recommended therapy for aGvHD?

1. Yes
2. No

Q10.4: In round 1 and 2, you agreed that ruxolitinib is the treatment that is most likely combined with ECP in chronic GvHD. Do you have any objections combining ECP with any other guideline recommended therapy for cGvHD?

1. Yes
2. No

Q11: Do you agree with the results from round 2 on the selection criteria for treating steroid-refractory GvHD (both acute and chronic) patients with ECP monotherapy?

	Strongly disagree	Disagree	Neither nor	Agree	Strongly agree
Rank 1: Low risk (e.g. skin involvement only or upper GI only)	❑	❑	❑	❑	❑
Rank 2: Contraindication for Ruxolitinib (e.g. thrombocytopenia)	❑	❑	❑	❑	❑

Q12: Which data generation strategy would you recommend Therakos/Mallinckrodt pursues in aGvHD?Please rank the suggestions below:

- Early use of ECP as monotherapy or in combination with steroids __________
- Proof of concept of ECP in prophylaxis __________
- Retrospective study in steroid-refractory aGvHD combination ECP and ruxolitinib __________
- Prospective study in steroid-refractory aGvHD

combination ECP and ruxolitinib __________

- Prospective study for efficacy of ECP in steroid-refractory aGvHD __________

Q12.1: Which other data generation on ECP would you recommend in aGvHD?

Q13: Which data generation strategy would you recommend Therakos/Mallinckrodt pursues in cGvHD?Please rank the suggestions below:

- Early use of ECP as monotherapy or in combination with steroids __________
- Retrospective study in steroid-refractory cGvHD combination ECP and ruxolitinib __________
- Prospective study in steroid-refractory cGvHD combination ECP and ruxolitinib __________
- Prospective study in steroid-refractory cGvHD combination ECP and belumosudil __________

- Prospective study for ECP in steroid-refractory cGvHD (single arm) __________

Which other data generation on ECP would you recommend in cGvHD?

Additional Results from The Delphi Study

Reasons for selecting ECP

Experts reached consensus (100% agreement for all) that the main reasons for selecting ECP as treatment for SR-GvHD are (1) its efficacy, (2) safety profile, and (3) steroid-sparing effect.

Combination of ECP with other GvHD therapies

Experts reached 100% agreement that the main reasons for a primary combination of ECP and ruxolitinib in SR-GvHD treatment include the potentially increased efficacy and that this combination is often used in severe GvHD. Increased efficacy was ranked more important than GvHD severity in SR-cGvHD and the inverse in SR-aGvHD.

Partial consensus was reached on the reasons for not adding ECP to ruxolitinib in SR-aGvHD; in order of importance, (1) inconvenience for the patient (91% agreement), (2) 'other reasons' (e.g. lack of venous access or low white blood cell count) (91% agreement), and (3) low efficacy (55% agreement). In SR-cGvHD patients, 91% of experts agreed that the main reasons for not adding ECP to ruxolitinib are (1) inconvenience for the patient followed by (2) organizational/logistic reasons (such as restricted hospital capacity).

Experts also agreed that the main reasons for stopping combination therapy in both SR-aGvHD and SR-cGvHD are, in order of importance, (1) the lack of efficacy (100% agreement), (2) inconvenience for patients (91% agreement), (3) side effects (aGvHD only; 82% agreement), and (3) ‘other reasons’ (e.g. failure to reduce steroid dosage) (cGvHD only; 91% agreement).

ECP as monotherapy

There is limited published data on ECP monotherapy for SR-GvHD and therefore experts were asked explorative questions which would require further clinical validation.

73% and 91% of experts agreed that ECP has the potential to be used as a monotherapy in SR-aGvHD and SR-cGvHD patients, respectively. 91% of experts agreed that the selection criteria for treating SR-GvHD patients with ECP monotherapy, in order of importance, are low-risk patients (e.g. those with skin involvement only or upper gastrointestinal involvement) and contraindication to ruxolitinib (e.g. thrombocytopenia, or high risk of infection).I

Appendix Figure 1: Overview of Delphi study results

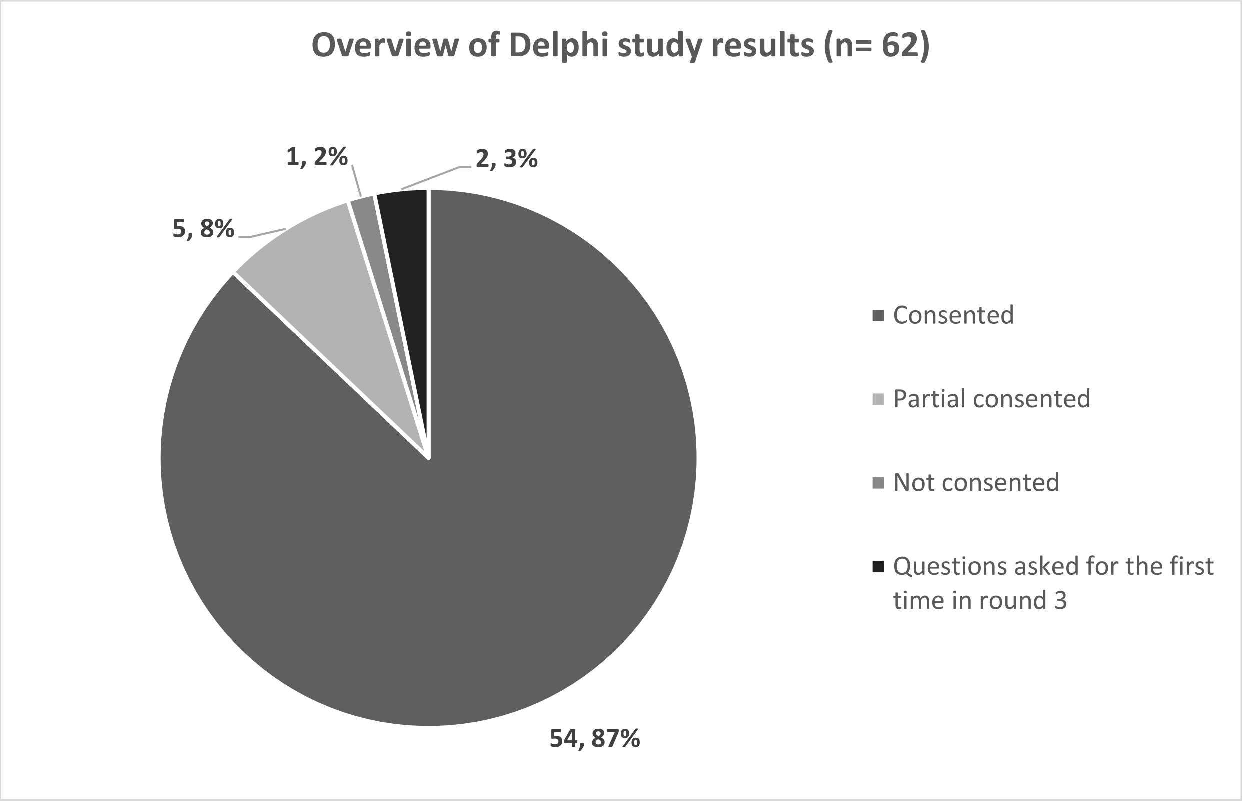

Footnotes: Of the 62 statements, 54 (87%) reached complete consensus whilst 5 (8%) reached partial consensus (i.e. some but not all sub-statements reached consensus) at the end of the three rounds. The remaining 3 (5%) statements did not reach consensus, of which 2 (3%) were asked for the first time during round 3. Of the 54 statements that reached complete consensus, 37 did so in round 2.

Appendix Table 1: Breakdown of level of consensus by theme

Topic	Total number of statements	aGvHD		cGvHD		Both aGvHD and cGvHD	
		Number of statements	Did all statements reach consensus?	Number of statements	Did all statements reach consensus?	Number of statements	Did all statements reach consensus?
**GvHD treatment goals and time to treatment**	4	2	Y	2	Y	0	N/A
**Reasons for selecting**	6	3	Y	3	Y	0	N/A

Topic	Total number of statements	aGvHD		cGvHD		Both aGvHD and cGvHD	
		Number of statements	Did all statements reach consensus?	Number of statements	Did all statements reach consensus?	Number of statements	Did all statements reach consensus?
**ECP or ruxolitinib**							
**Efficacy and challenges of ECP treatment**	2	0	N/A	0	N/A	2	Partial (Q4 of round 2)
**Treatment duration and**	4	2	Partial (Q3.1 of round 3)	2	No consensus	0	N/A

Topic	Total number of statements	aGvHD		cGvHD		Both aGvHD and cGvHD	
		Number of statements	Did all statements reach consensus?	Number of statements	Did all statements reach consensus?	Number of statements	Did all statements reach consensus?
**schedule of ECP**					(Q1.2 of round 3)		
**Combination of ECP with other GvHD therapies**	16	8	Partial (Q21.1 of round 2, Q25.1 of round 2)	8	Partial (Q25.2 of round 2)	0	N/A
**Sequential treatment**	4	2	Y	2	Y	0	N/A

Topic	Total number of statements	aGvHD		cGvHD		Both aGvHD and cGvHD	
		Number of statements	Did all statements reach consensus?	Number of statements	Did all statements reach consensus?	Number of statements	Did all statements reach consensus?
**Treatment switching (from ruxolitinib to ECP and vice-versa)**	2	1	Y	1	Y	0	N/A
**Reducing and stopping**	8	4	Y	4	Y	0	N/A

Topic	Total number of statements	aGvHD		cGvHD		Both aGvHD and cGvHD	
		Number of statements	Did all statements reach consensus?	Number of statements	Did all statements reach consensus?	Number of statements	Did all statements reach consensus?
**monotherapy**							
**Treatment cessation**	6	3	Y	3	Y	0	N/A
**Ruxolitinib refractoriness**	3	1	Y	1	Y	1	Y

Topic	Total number of statements	aGvHD		cGvHD		Both aGvHD and cGvHD	
		Number of statements	Did all statements reach consensus?	Number of statements	Did all statements reach consensus?	Number of statements	Did all statements reach consensus?
**ECP use in non-SR patients**	2	1	Y	1	Y	0	N/A
**ECP monotherapy**	3	1	Y	1	Y	1	Y
**Data generation strategies**	2	1	N/A - First time asked in round 3	1	N/A - First time asked in round 3	0	N/A

**Topic**	**Total number of statements**	**aGvHD**		**cGvHD**		**Both aGvHD and cGvHD**	
		**Number of statements**	**Did all statements reach consensus?**	**Number of statements**	**Did all statements reach consensus?**	**Number of statements**	**Did all statements reach consensus?**
**Overall**	62	29	Partial consensus: Q3.1 of round 3 (SR-aGvHD treatment schedule), Q21.1 of round 2	29	Partial consensus: Q25.2 of round 2 (Therapies to combine with ECP in cGvHD)	4	Partial consensus: Q4 of round 2 (Organ with most improvement following

Topic	Total number of statements	aGvHD		cGvHD		Both aGvHD and cGvHD	
		Number of statements	Did all statements reach consensus?	Number of statements	Did all statements reach consensus?	Number of statements	Did all statements reach consensus?
			(Reasons for not adding ECP to ruxolitinib in SR-aGvHD) and Q25.1 of round 2		No consensus: Q1.2 of round 3(SR-cGvHD treatment schedule)		ECP treatment)

Topic	Total number of statements	aGvHD		cGvHD		Both aGvHD and cGvHD	
		Number of statements	Did all statements reach consensus?	Number of statements	Did all statements reach consensus?	Number of statements	Did all statements reach consensus?
			(Therapies to combine with ECP in aGvHD)				

Abbreviations: aGvHD: acute graft-versus-host disease; cGvHD: chronic graft-versus-host disease; ECP: extracorporeal photopheresis; GvHD: graft-versus-host disease; N/A: not applicable; SR: steroid-refractory; Y: yes.

Appendix Table 2: Proposed definition of ruxolitinib refractoriness

Question to the experts	Statements	Agreement %
Would you agree on the following definition of ruxolitinib refractoriness in aGvHD?	Progression of GvHD compared to baseline in any organ after at least 5 days of treatment with ruxolitinib, based either on objective increase in stage/grade, or new organ involvement	91
	Lack of improvement in GvHD (PR or better) compared to baseline within 5 – 10 days of treatment with ruxolitinib	
	Loss of response, defined as objective worsening of GvHD determined by increase in stage, grade or new organ involvement at any time after initial improvement	
How do you determine that a cGvHD patient is refractory to ruxolitinib? Do	Progression of GvHD compared to baseline after 1 – 2 weeks of treatment with ruxolitinib, based either on objective increase in stage/grade, or new organ involvement	100

you agree on these criteria, resulting from round 1?	Lack of improvement in GvHD (PR or better) compared to baseline after 2 – 3 months of treatment with ruxolitinib	82
	Loss of response, defined as objective worsening of GvHD determined by increase in severity or new organ involvement at any time after initial improvement	100

Abbreviations: aGvHD: acute graft-versus-host disease; cGvHD: chronic graft-versus-host disease; GvHD: graft-versus-host disease; PR: partial response.

Appendix Table 3: Summary of results from Delphi study rounds

Question	Consensus	Round 1 Results	Round 2 Results	Round 3 Results
Q1.1 What are your top treatment goals in treating steroid refractory aGvHD?	Yes	Average rank 1.55: Achieve complete response<br>Average rank 1.82: Improve length of survival<br>Average rank 3.12: Achieve partial response<br>Average rank 3.38: Improve quality of life	Q1.1<br>Rank 1: Achieve complete response<br>Rank 2: Improve length of stay<br>Rank 3: Achieve partial response<br><br>*(consensus)*	-
Q1.2: What are your top treatment goals in treating steroid refractory cGvHD?	Yes	Average rank 1.78: Achieve complete response<br>Average rank 2.33: Improve length of survival	Q1.2<br>Rank 1: Achieve complete response<br>Rank 2: Improve length of survival	-

Question	Consensus	Round 1 Results	Round 2 Results	Round 3 Results
		Average rank 2.4: Improve quality of life<br>Average rank 2.78: Achieve partial response	Rank 3: Achieve partial response<br><br>*(consensus)*	
Q2.1: Which factors influence you to select ECP as a treatment in steroid refractory aGvHD patients?	Yes	Average rank 1.64: Efficacy of ECP<br>Average rank 2.09: Safety profile<br>Average rank 3.09: Steroid-sparing effect<br>Average rank 4.1: Possibility to comb. With other IS therapies<br>Average rank 5: Patients' adherence to treatment	Q2.1<br>Rank 1: Efficacy of ECP<br>Rank 2: Safety profile<br>Rank 3: Steroid-sparing effect<br><br>*(consensus)*	-
Q2.2: Which factors influence you to select ECP as a treatment in steroid refractory cGvHD patients?	Yes	Average rank 1.45: Efficacy of ECP<br>Average rank 2.55: Safety profile	Q2.2<br>Rank 1: Efficacy of ECP<br>Rank 2: Safety profile	-

Question	Consensus	Round 1 Results	Round 2 Results	Round 3 Results
		Average rank 2.64: Steroid-sparing effect<br>Average rank 3.22: Possibility to comb. with other IS therapies<br>Average rank 5: Patients' adherence to treatment	Rank 3: Steroid-sparing effect<br><br>*(consensus)*	
Q3.1: When treating steroid refractory aGvHD patients with ECP alone* but not with ruxolitinib - which treatment schedules do you apply?<br><br>* +/- steroids and/or CNI	Partial	Median treatment schedule 1: 2.5 procedures per week, 82% yes on consecutive days, weekly, duration 4 weeks<br>Median treatment schedule 2: 1 procedure per week, 63% yes on consecutive days, weekly, duration 8 weeks	Q3.1<br>Treatment schedule 1: 2-3 ECP procedures per week on consecutive days weekly for 4 weeks<br>Treatment schedule 2: 1-2 ECP procedures per week on consecutive days weekly for 8 weeks	Q3.1<br>Treatment schedule 1: 2-3 ECP procedures per week on consecutive days weekly for 4 weeks<br>Treatment schedule 2: 2 ECP procedures per week at least every two weeks for

**Question**	**Consensus**	**Round 1 Results**	**Round 2 Results**	**Round 3 Results**
		<u>Median treatment schedule 3</u>: 0.5 procedures per week, 72% yes on consecutive days, monthly, duration 8 weeks	<u>Treatment schedule 3:</u> 1-2 ECP procedures per week on consecutive days monthly for 8 weeks<br><br>*(partial consensus)*	approximately 8 weeks (2 month)<br><u>Treatment schedule 3:</u> 2 ECP procedures per week at least every month for approximately 8 weeks (2 month)<br><br>*(partial consensus)*
Q3.2: When treating steroid refractory cGvHD patients with ECP alone* - which treatment schedules do you apply? *+/- steroids and/or CNI	No	<u>Median treatment schedule 1</u>: 2 procedures per week, 80% yes, weekly, duration 9 weeks<br><u>Median treatment schedule 2:</u> 1.5 procedures per weeks, 78% yes on	Q3.2<br><u>Treatment schedule 1:</u> 2 ECP procedures per week on consecutive days weekly for 9 weeks	Q1.2<br><u>Treatment schedule 1:</u> 2 ECP procedures per week for approximately 9 weeks<br><u>Treatment schedule 2:</u> 2 ECP procedures per week, at least

Question	Consensus	Round 1 Results	Round 2 Results	Round 3 Results
		consecutive days, weekly, duration 10 weeks<br>Median treatment schedule 3: 1.5 procedures per weeks, 78% yes on consecutive days, monthly, duration 20 weeks	Treatment schedule 2: 1-3 ECP procedures per week on consecutive days weekly for 10 weeks<br>Treatment schedule 3: 1-2 ECP procedures per week on consecutive days monthly for 5 months<br><br>*(no consensus)*	every two weeks for approximately 10 weeks<br>Treatment schedule 3: 1-2 ECP procedures per week at least monthly for approximately 20 weeks (5 month)<br><br>*(no consensus)*
Q4.1: Would you ever continue ECP treatment despite a lack of visible clinical improvements?	-	73% Yes<br>27% No	-	-

Question	Consensus	Round 1 Results	Round 2 Results	Round 3 Results
Q4.2: What are your reasons for continuing ECP treatments?	-	Average rank 1.33: Possibility to combine ECP with other immunosuppressive treatments<br>Average rank 2: ECP's safety profile<br>Average rank 2.5: Improvement of quality of life despite clinical improvements<br>Average rank 4: Patients' preferences<br><br>*(only asked in round 1)*	-	-
Q5: In which organs do you see the most improvements following ECP treatment?	Partial	Average rank 1.18: Skin<br>Average rank 2.33: Oral mucosa<br>Average rank 7.41: Gut<br>Average rank 5: Musculoskeletal<br>Average rank 5.2: Fascia	Q4<br>Rank 1: Skin<br>Rank 2: Oral mucosa	-

Question	Consensus	Round 1 Results	Round 2 Results	Round 3 Results
		Average rank 5.71: Liver<br>Average rank 6.17: Salivary glands<br>Average rank 6.33: Eyes<br>Average rank 7.33: Genital mucosa<br>Average rank 7.62: Lung<br>Average rank 9.33: Kidney	Rank 3: Gut, musculoskeletal, fascia, liver, salivary glands, eyes, genital mucosa and lung<br>Rank 4: Kidney<br><br>*(partial consensus)*	
Q6.1: In your own practice, what is the percentage of patients with steroid refractory aGvHD that receive various treatments as per described below?	Yes	Regimen containing neither ruxolitinib nor ECP: 6%<br>Regimen containing ruxolitinib, but no ECP: 49%<br>Regimen containing ECP, but no ruxolitinib: 25%<br>Regimen containing both, ruxolitinib and ECP: 20%	Q5.1<br>○ Approx. 49% of aGvHD patients receive a regimen containing ruxolitinib but no ECP<br>○ Approx 25% of aGvHD patients receive a regimen containing ECP, but no ruxolitinib	-

Question	Consensus	Round 1 Results	Round 2 Results	Round 3 Results
			○ Approx. 20% of aGvHD patients receive a regimen containing both ECP and ruxolitinib<br>○ Approx. 6% of aGvHD patients receive neither ECP nor ruxolitinib<br><br>*(consensus)*	
Q6.2: In your own practice, what is the percentage of patients with steroid refractory cGvHD that receive various treatments as per described below?	Yes	Regimen containing neither ruxolitinib nor ECP: 12%<br>Regimen containing ruxolitinib, but no ECP: 36%	Q5.2<br>○ Approx. 36% of cGvHD of patients receive a regimen containing ruxolitinib, but no ECP	Q5.2<br>○ Approx. 30%-40% of cGvHD patients receive a regimen containing

Question	Consensus	Round 1 Results	Round 2 Results	Round 3 Results
		Regimen containing ECP, but no ruxolitinib: 33%<br>Regimen containing both, ruxolitinib and ECP: 19%	○ Approx. 33% of cGvHD of patients receive a regimen containing ECP, but no ruxolitinib<br>○ Approx. 19% of cGvHD of patients receive a regimen containing both ECP and ruxolitinib<br>○ Approx. 12% of cGvHD patients receive neither ECP nor ruxolitinib<br><br>*(no consensus)*	ruxolitinib, but no ECP<br>○ Approx. 20%-30% of cGvHD patients receive a regimen containing ECP, but not ruxolitinib<br>○ Approx. 10%-20% of cGvHD patients receive a regimen containing both ECP and ruxolitinib<br>○ Approx. 10%-20% of cGvHD patients

Question	Consensus	Round 1 Results	Round 2 Results	Round 3 Results
				receive neither ECP nor ruxolitinib<br><br>*(consensus)*
Q6.3: Of the steroid refractory aGvHD patients receiving a combination therapy with ECP and ruxolitinib, what percentage start both treatments simultaneously?	Yes	Percentage of initial ECP-ruxolitinib combinations: 16%<br>Percentage of sequential ECP-ruxolitinib combinations: 84%	Q6.1<br>○ Approx. 16% of aGvHD patients receiving a combination therapy both ECP and ruxolitinib simultaneously<br><br>*(consensus)*	-
Q6.4: Of the steroid refractory cGvHD patients receiving a combination therapy	Yes	Percentage of initial ECP-ruxolitinib combinations: 10%	Q6.2	-

Question	Consensus	Round 1 Results	Round 2 Results	Round 3 Results
with ECP and ruxolitinib, what percentage start both treatments simultaneously?		Percentage of sequential ECP-ruxolitinib combinations: 90%	○ Approx. 10% of cGvHD patients receiving a combination therapy both ECP and ruxolitinib simultaneously<br><br>*(consensus)*	
Q7: What are the main reasons for choosing ECP, but no ruxolitinib, in steroid refractory aGvHD patients?	Yes	Please see Appendix Table 2 below<br><br>Summarised options for the ranking in round 2:<br>○ Contraindication and safety profile of ruxolitinib<br>○ Ruxolitinib failure	Q7.1<br><u>Average rank</u> 1.27: Contraindication and safety profile of ruxolitinib<br><u>Average rank</u> 2.18: Ruxolitinib failure	Q7.1<br><u>Rank 1:</u> Contraindication and safety profile of ruxolitinib<br><u>Rank 2:</u> Ruxolitinib failure<br><u>Rank 3:</u> Safety profile of ECP and high efficacy of ECP

Question	Consensus	Round 1 Results	Round 2 Results	Round 3 Results
		○ High efficacy of ECP, especially in patients with skin involvement<br>○ Safety profile of ECP	Average rank 2.64: High efficacy in patients with skin involvement<br>Average rank 2.64: safety profile of ECP<br><br>*(first time asked)*	(especially in patients with skin involvement)<br><br>*(consensus)*
Q7: What are the main reasons for choosing ruxolitinib, but no ECP, in steroid refractory aGvHD patients?	Yes	Please see Appendix Table 2 below<br><br>Summarised options for the ranking in round 2:<br>○ High efficacy, especially in patients with gastrointestinal involvement<br>○ Regulatory reasons (e.g. EMA approval)	Q7.2<br>Average rank 1.27: High efficacy especially in patients with gastrointestinal involvement<br>Average rank 1.91: regulatory reasons (e.g. EMA approval)<br>Average rank 2.36: Venous access not necessary	Q7.2<br>Rank 1: High efficacy, especially in patients with gastrointestinal involvement<br>Rank 2: regulatory reasons (e.g. EMA approval)<br>Rank 3: Venous access not necessary

**Question**	**Consensus**	**Round 1 Results**	**Round 2 Results**	**Round 3 Results**
		○ Venous access not necessary	*(first time asked)*	*(consensus)*
Q7: What are the main reasons for choosing the combination therapy of ECP and ruxolitinib in steroid refractory aGvHD patients?	Yes	Please see Appendix Table 2 below<br><br>Summarised options for the ranking in round 2:<br>○ Increased efficacy<br>○ Severe case	Q7.3<br><u>Average rank</u> 1.36: Severe cases<br><u>Average rank</u> 1.45: Increased efficacy<br><br>*(first time asked)*	Q7.3<br><u>Rank 1:</u> Severe cases<br><u>Rank 2:</u> Increased efficacy<br><br>*(consensus)*
Q7: What are the main reasons for choosing ECP, but no ruxolitinib, in steroid refractory cGvHD patients?	Yes	Please see Appendix Table 3 below<br><br>Summarised options for the ranking in round 2:<br>○ Contraindication to ruxolitinib<br>○ Ruxolitinib failure	Q8.1<br><u>Average rank</u> 1.27: Contraindication to ruxolitinib<br><u>Average rank</u> 2.27: Ruxolitinib failure<br><u>Average rank</u> 2.27: Efficacy, esp. in patients with skin	Q8.1<br><u>Rank 1:</u> Contraindication to ruxolitinib<br><u>Rank 2:</u> Ruxolitinib failure<br><u>Rank 3:</u> Efficacy, esp. in patients with skin involvement and for steroid sparing effect

**Question**	**Consensus**	**Round 1 Results**	**Round 2 Results**	**Round 3 Results**
		○ Efficacy, esp. in patients with skin involvement and for steroid spearing effect<br>○ Safety profile	involvement and for steroid spearing effect<br>Average rank 2.82: safety profile<br><br>*(first time asked)*	Rank 4: Safety profile<br>*(consensus)*
Q7.2: What are the main reasons for choosing ruxolitinib, but no ECP, in steroid refractory cGvHD patients?	Yes	Please see Appendix Table 3 below<br><br>Summarised options for the ranking in round 2:<br>○ High efficacy, esp. in patients with gastrointestinal involvement<br>○ Regulatory reasons (e.g. EMA approval)<br>○ Patient preferences	Q8.2<br>Average rank 1.18: High efficacy, esp. in patients with gastrointestinal involvement<br>Average rank 1.55: Regulatory reasons (e.g. EMA approval)<br>Average rank 2.27: Patient preferences<br><br>*(first time asked)*	Q8.2<br>Rank 1: High efficacy, esp. in patients with gastrointestinal involvement<br>Rank 2: Regulatory reasons (e.g. EMA approval)<br>Rank 3: Patient preferences<br><br>*(consensus)*

**Question**	**Consensus**	**Round 1 Results**	**Round 2 Results**	**Round 3 Results**
Q7.3: What are the main reasons for choosing the combination therapy of ECP & ruxo in steroid-refractory cGvHD?	Yes	Please see Appendix Table 3 below<br><br>Summarised options for the ranking in round 2:<br>○ Increased efficacy<br>○ Severe cases	Q8.3<br>Average rank 1.36: Increased efficacy<br>Average rank 1.45: Severe cases<br><br>*(first time asked)*	Q8.3<br>Rank 1: Increased efficacy<br>Rank 2: Severe cases<br><br>*(consensus)*
Q8: Based on your practice, what is the average time between steroid refractory diagnosis and treatment start for the treatments below?	Yes	Aggregated data:<br>**Median time between steroid refractory diagnosis and treatment start – aGvHD**<br>ECP, but no ruxolitinib: 3 days<br>Ruxolitinib: 3.5 days<br>ECP & ruxolitinib: 9 days<br><br>Aggregated data:	Q9.1<br>○ In aGvHD patients treated with ECP, the median time between SR diagnosis and treatment is 3 days<br>○ In aGvHD patients treated with ruxolitinib, the median time	Q9.1<br>○ In aGvHD patients treated with ruxolitinib, the time between SR diagnosis and treatment start is maximum 4 days

Question	Consensus	Round 1 Results	Round 2 Results	Round 3 Results
		**Median time between steroid refractory diagnosis and treatment start – cGvHD**<br>ECP, but no ruxolitinib: 14 days<br>Ruxolitinib: 10 days<br>ECP & ruxolitinib: 30 days	between SR diagnosis and treatment start is 3-4 days<br>○ In aGvHD patients treated with both ECP and ruxolitinib, the median time be tween SR diagnosis and treatment start is 9 days<br>*(partial consensus)*<br>Q9.1	*(consensus)*

Question	Consensus	Round 1 Results	Round 2 Results	Round 3 Results
			○ In cGvHD patients treated with ECP, the median time between SR diagnosis and treatment start is 14 days<br>○ In cGvHD patients treated with ruxolitinib, the median time between SR diagnosis and treatment start is 10 days<br>○ In cGvHD patients treated with both ECP and ruxolitinib, the	

Question	Consensus	Round 1 Results	Round 2 Results	Round 3 Results
			median time between SR diagnosis and treatment start is 30 days<br><br>*(consensus)*	
Q9.1: Based on your practice and depending on the applied treatment what is the average treatment duration of ECP/ruxolitinib in steroid refractory aGvHD in the following scenarios?	Yes	Aggregated data:<br>**Median duration – aGvHD**<br>ECP without ruxolitinib: 20 weeks<br>Ruxolitinib without ECP: 16 weeks<br>ECP with ruxolitinib: 25 weeks<br>Ruxolitinib with ECP: 12 weeks<br><br>**Median duration – cGvHD**<br>ECP without ruxolitinib: 48 weeks	Q10.1<br>○ The average treatment duration of ECP in aGvHD patients is 20 days<br>○ The average treatment duration of ruxolitinib in aGvHD patients is 16 days	Q10.1<br>○ The average treatment duration of ECP in aGvHD patients is 4-6 months<br>○ The average treatment duration of ruxolitinib in

Question	Consensus	Round 1 Results	Round 2 Results	Round 3 Results
		Ruxolitinib without ECP: 50 weeks<br>ECP with ruxolitinib: 50 weeks<br>Ruxolitinib with ECP: 40 weeks	○ The average treatment duration of ECP in combination with ruxolitinib in aGvHD patients is 25 days<br>○ The average treatment duration of ruxolitinib in combination with ECP in aGvHD patients is 12 days<br><br>*(no consensus)*<br><br>Q10.2	aGvHD patients is 3-5 months<br>○ The average treatment duration of ECP in combination with ruxolitinib in aGvHD patients is 4-6 months<br>○ The average treatment duration of ruxolitinib in combination with ECP in aGvHD

**Question**	**Consensus**	**Round 1 Results**	**Round 2 Results**	**Round 3 Results**
			○ The average treatment duration of ECP in cGvHD patients is 48 days<br>○ The average treatment duration of ruxolitinib in cGvHD patients is 50 days<br>○ The average treatment duration of ECP in a combination with ruxolitinib in cGvHD patients is 50 days<br>○ The average treatment duration of ruxolitinib	patients is 3-5 month<br><br>*(consensus)*<br>Q10.2<br>○ The average treatment duration of ECP in cGvHD patients is 4-6 months<br>○ The average treatment duration of ruxolitinib in cGvHD patients is 3-5 months

Question	Consensus	Round 1 Results	Round 2 Results	Round 3 Results
			in a combination with ECP in cGvHD patients is 40 days<br><br>*(no consensus)*	○ The average treatment duration of ECP in a combination with ruxolitinib in cGvHD patients is 4-6 months<br>○ The average treatment duration of ruxolitinib in a combination with ECP in cGvHD patients is 3-5 months

Question	Consensus	Round 1 Results	Round 2 Results	Round 3 Results
				(consensus)
Q10.1- Q10.4: What percentage of patients that are treated with ECP switch from ECP to ruxolitinib?	Yes	Aggregated data:<br>**aGvHD**<br>ECP → ruxolitinib: 20.3%<br>Ruxolitinib → ECP: 18%<br>ECP → combination with ruxolitinib: 29.7%<br>Ruxolitinib → combination with ECP: 24.1%<br><br>**cGvHD**<br>ECP → ruxolitinib: 21%<br>Ruxolitinib → ECP: 22%<br>ECP → combination with ruxolitinib: 28.1%	Q11.1<br>○ On average, 20% of aGvHD patients treated with ECP switch to ruxolitinib<br>○ On average, 18% of aGvHD patients treated with ruxolitinib switch to ECP<br>○ On average, 30% of aGvHD patients treated with ECP switch to a combination therapy of ECP and ruxolitinib	-

Question	Consensus	Round 1 Results	Round 2 Results	Round 3 Results
		Ruxolitinib → combination with ECP: 23.5%	○ On average, 24% of aGvHD patients treated with ruxolitinib switch to a combination therapy of ruxolitinib and ECP<br><br>*(consensus)*<br><br>Q11.2<br>○ On average, 21% of cGvHD patients treated with ECP switch to ruxolitinib	

Question	Consensus	Round 1 Results	Round 2 Results	Round 3 Results
			○ On average, 22% of cGvHD patients treated with ruxolitinib switch to ECP<br>○ On average, 28% of cGvHD patients treated with ECP switch to a combination therapy of ECP and ruxolitinib<br>○ On average, 24% of cGvHD patients treated with ruxolitinib switch to a combination therapy of ruxolitinib and ECP	

Question	Consensus	Round 1 Results	Round 2 Results	Round 3 Results
			(consensus)	
Q11.1: Depending on the applied treatment: In your practice, what is the percentage of steroid refractory GvHD patients where steroids could be reduced by at least 50%?	Yes	Aggregated data:<br>**aGvHD**<br>ECP without ruxolitinib: 50%<br>Ruxolitinib without ECP: 52.5%<br>Combination treatment: 80%<br><br>**cGvHD**<br>ECP without ruxolitinib: 60%<br>Ruxolitinib without ECP: 65%<br>Combination treatment: 80%	Q12.1<br>○ In 50% of aGvHD patients treated with ECP, steroids could be reduced by at least 50%<br>○ In 53% of aGvHD patients treated with ruxolitinib, steroids could be reduced by at least 50%<br>○ In 80% of aGvHD patients treated with	-

Question	Consensus	Round 1 Results	Round 2 Results	Round 3 Results
			the combination of ECP and ruxolitinib, steroids could be reduced by at least 50%. *(consensus)*<br><br>Q12.2<br>○ In 60% of cGvHD patients treated with ECP, steroids could be reduced by at least 50%<br>○ In 65% of cGvHD patients treated with ruxolitinib, steroids	

Question	Consensus	Round 1 Results	Round 2 Results	Round 3 Results
			could be reduced by at least 50%<br>○ In 80% of cGvHD patients treated with the combination of ECP and ruxolitinib, steroids could be reduced by at least 50%.<br><br>*(consensus)*	
Q11.2: How long would it take before you would be able to implement a 50% reduction of steroid dosage?	Yes	Aggregated data:<br>**aGvHD**<br>ECP without ruxolitinib: 29 days<br>Ruxolitinib without ECP: 23 days<br>Combination therapy: 15 days	Q13.1<br>○ On average, it takes 29 days to implement a 50% reduction of steroid dosage in	-

Question	Consensus	Round 1 Results	Round 2 Results	Round 3 Results
		**cGvHD**<br>ECP without ruxolitinib: 56 days<br>Ruxolitinib without ECP: 51 days<br>Combination therapy: 46 days	cGvHD patient treated with ECP<br>○ On average, it takes 23 days to implement a 50% reduction of steroid dosage in aGvHD patients treated with ruxolitinib<br>○ On average, it takes 15 days to implement a 50% reduction of steroid dosage in aGvHD patients with the combination of ECP and ruxolitinib	

Question	Consensus	Round 1 Results	Round 2 Results	Round 3 Results
			(consensus)<br><br>Q13.2<br>○ On average, it takes 56 days to implement a 50% reduction of steroid dosage in cGvHD patient treated with ECP<br>○ On average, it takes 51 days to implement a 50% reduction of steroid dosage in	

Question	Consensus	Round 1 Results	Round 2 Results	Round 3 Results
			cGvHD patients treated with ruxolitinib<br>○ On average, it takes 46 days to implement a 50% reduction of steroid dosage in cGvHD patients with the combination of ECP and ruxolitinib<br><br>*(consensus)*	
Q11.3: Depending on the applied treatment: What is the percentage of steroid refractory GvHD patients in your practice where steroid treatment could be stopped completely?	Yes	Aggregated data:<br>**aGvHD**<br>ECP without ruxolitinib: 50%<br>Ruxolitinib without ECP: 51%	Q14.1<br>○ On average, steroid treatment could be stopped in 50% of	-

Question	Consensus	Round 1 Results	Round 2 Results	Round 3 Results
		Combination therapy: 70%<br><br>**cGvHD**<br>ECP without ruxolitinib: 41%<br>Ruxolitinib without ECP: 40%<br>Combination therapy: 60%	aGvHD patients treated with ECP<br>○ On average, steroid treatment could be stopped in 51% of aGvHD patients with ruxolitinib<br>○ On average, steroid treatment could be stopped in 70% of aGvHD patient treated with the combination of ECP and ruxolitinib<br><br>*(consensus)*	

Question	Consensus	Round 1 Results	Round 2 Results	Round 3 Results
			Q14.2<br>○ On average, steroid treatment could be stopped in 41% of cGvHD patients treated with ECP<br>○ On average, steroid treatment could be stopped in 40% of cGvHD patients with ruxolitinib<br>○ On average, steroid treatment could be stopped in 60% of	

Question	Consensus	Round 1 Results	Round 2 Results	Round 3 Results
			cGvHD patient treated with the combination of ECP and ruxolitinib<br><br>*(consensus)*	
Q11.4: How long would it take before you would be able to stop steroid treatment completely?	Yes	Aggregated data:<br>**aGvHD**<br>ECP without ruxolitinib: 84 days<br>Ruxolitinib without ECP: 75 days<br>Combination therapy: 75 days<br><br>**cGvHD**<br>ECP without ruxolitinib: 165 days<br>Ruxolitinib without ECP: 180 days<br>Combination therapy: 113 days	Q15.1<br>○ On average, it takes 84 days to completely stop steroid treatment in aGvHD patients treated with ECP<br>○ On average, it takes 75 days to completely stop steroid treatment in	Q15.1<br>○ On average, it takes 2-3 months to completely stop steroid treatment in aGvHD patients treated with ECP<br>○ On average, it takes 2-3 months to completely stop

Question	Consensus	Round 1 Results	Round 2 Results	Round 3 Results
			aGvHD patients treated with ruxolitinib<br>○ On average, it takes 75 days to completely stop steroid treatment in aGvHD patients treated with the combination of ECP and ruxolitinib<br><br>*(no consensus)*<br><br>Q15.2<br>○ On average, it takes 5.5 month to completely stop steroid	steroid treatment in aGvHD patients treated with ruxolitinib<br>○ On average, it takes 2-3 month to completely stop steroid treatment in aGvHD patients treated with the combination of ECP and ruxolitinib<br><br>*(consensus)*

Question	Consensus	Round 1 Results	Round 2 Results	Round 3 Results
			treatment in cGvHD patients treated with ECP<br>○ On average, it takes 6 months to completely stop steroid treatment in cGvHD patients treated with ruxolitinib<br>○ On average, it takes 4 months to completely stop steroid treatment in cGvHD patients treated with the combination of ECP and ruxolitinib	

Question	Consensus	Round 1 Results	Round 2 Results	Round 3 Results
			(consensus)	
Q12.1: How do you determine that an aGvHD patient is refractory to ruxolitinib? Please rank the criteria according to their relative importance	Yes	Average rank 1.44: I apply the definition suggested by Mothy *et al.* 2020<br>Average rank 2: Progression in any organ within 3, 4 or 5 days of therapy onset with ruxolitinib<br>Average rank 2.17: Incomplete response after more than 28 days of ruxolitinib<br>Average rank 2.5: Failure to improve within 5 to 7 days of treatment initiation	Q16.1<br>In the following, two sets of criteria for the definition of ruxolitinib refractoriness in aGvHD patients are presented. Please choose which of these sets you fits your current practice and you align with?<br><br>Set 1 - Mohty *et al.* 2020<br>Progression of GvHD compared to baseline after at least 5 to 10 days of treatment with	Q16.1<br>Would you agree on the following definition of ruxolitinib refractoriness in aGvHD?<br>○ Progression of GvHD compared to baseline in any organ after at least 5 days of treatment with ruxolitinib, based either on objective increase in

Question	Consensus	Round 1 Results	Round 2 Results	Round 3 Results
			ruxolitinib, based either on objective increase in stage/grade, or new organ involvement;- Lack of improvement in GvHD (PR or better) compared to baseline after at least 14 days of treatment with ruxolitinib; or- Loss of response, defined as objective worsening of GvHD determined by increase in stage, grade or new organ involvement at any time after initial improvement.	stage/grade, or new organ involvement<br>○ Lack of improvement in GvHD (PR or better) compared to baseline within 5-10 days of treatment with ruxolitinib<br>○ Loss of response, defined as objective worsening of GvHD determined by increase in stage, grade or new organ involvement at any

Question	Consensus	Round 1 Results	Round 2 Results	Round 3 Results
			Set 2 - Modified criteria based on answers from round 1:- Progression of GvHD compared to baseline in any organ after at least 3 to 5 days of treatment with ruxolitinib, based either on objective increase in stage/grade, or new organ involvement;- Lack of improvement in GvHD (PR or better) compared to baseline within 5-7 days of treatment with ruxolitinib;- Loss of response, defined as objective worsening of GvHD determined	time after initial improvement. 91% agreed<br><br>*(consensus)*

Question	Consensus	Round 1 Results	Round 2 Results	Round 3 Results
			by increase in stage, grade or new organ involvement at any time after initial improvement.<br><br>Set 1: Mothy *et al.*: 73%<br>Set 2 Modified criteria based on answers from round one: 73%<br><br>*(no consensus)*	
Q12.2: How do you determine that a cGvHD patient is refractory to ruxolitinib? Please rank the following criteria according to their relative importance	Yes	<u>Average rank</u> 1: Progression of GvHD while on ruxolitinib for 1-2 weeks<br><u>Average rank</u> 1.33: I use a different definition for ruxolitinib-refractory (please indicate)<br>Given alternatives by experts:	Q16.2<br>How do you determine that a cGvHD patient is refractory to ruxolitinib? Do you agree on these criteria, resulting from round 1?	-

Question	Consensus	Round 1 Results	Round 2 Results	Round 3 Results
		○ Progression while on ruxo for 2-4 weeks, stable GvHD while on ruxo for 2 months, any new organ localization during ruxo<br>○ No improvement after 3 months<br>○ Stable GvHD while on ruxo for 3 months<br>Average rank 1.88: stable GvHD while on ruxolitinib for 1-2 months	○ Progression of GvHD compared to baseline after 1-2 weeks of treatment with ruxolitinib, based either on objective increase in stage/grade, or new organ involvement<br>○ Lack of improvement in GvHD (PR or better) compared to baseline after 2-3 month of treatment with ruxolitinib	

Question	Consensus	Round 1 Results	Round 2 Results	Round 3 Results
			○ Loss of response, defined as objective worsening of GvHD determined by increase in severity or new organ improvement<br><br>*(consensus)*	
Q12.3: In your clinical practice, what is the average treatment time on ruxolitinib before ruxolitinib refractoriness is established?	Yes	Aggregated data:<br>aGvHD: 14 days<br>cGvHD: 60 days	Q17.1<br>○ It takes aGvHD patients, on average, 14 days on ruxolitinib before ruxolitinib refractoriness is established	-

**Question**	**Consensus**	**Round 1 Results**	**Round 2 Results**	**Round 3 Results**
			○ It takes cGvHD patients, on average, 60 days on ruxolitinib before ruxolitinib refractoriness is established<br><br>*(consensus)*	
Q13.1: When treating steroid refractory aGvHD patients with ECP, what would be reasons for stopping ECP treatment?	Yes	Average rank 1.18: Lack of efficacy<br>Average rank 2: Inconvenience of the patient<br>Average rank 2: Side effects	Q18.1<br>Rank 1: Lack of efficacy<br>Rank 2: Inconvenience for the patient<br>Rank 3: Side effects<br><br>*(consensus)*	-

Question	Consensus	Round 1 Results	Round 2 Results	Round 3 Results
Q13.2: When treating steroid refractory cGvHD patients with ECP, what would be reasons for stopping ECP treatment?	Yes	Average rank 1.18: Lack of efficacy<br>Average rank 2: Inconvenience for the patient<br>Average rank 2.5: Another reason, namely:<br>Given alternatives by experts:<br>○ Loss of venous access<br>○ Failure to reduce steroid dose<br>Average rank 2.75: Side effects	Q18.2<br>Rank 1: Lack of efficacy<br>Rank 2: Inconvenience<br>Rank 3: Other reasons, e.g. Loss of venous access or failure to reduce steroid dosage<br><br>*(consensus)*	-
Q14.1: When treating steroid refractory aGvHD patients with ruxolitinib, what would be reasons for stopping ruxolitinib treatment?	Yes	Average rank 1.36: Lack of efficacy<br>Average rank 1.6: Side effects<br>Average rank 2: Another reason, namely:<br>Given alternatives by experts:	Q19.1<br>Rank 1: Lack of efficacy<br>Rank 2: Side effects	-

**Question**	**Consensus**	**Round 1 Results**	**Round 2 Results**	**Round 3 Results**
		○ Failure stop/reduce steroid dose<br>Average rank 3: Inconvenience for the patient	Rank 3: Other reasons, e.g. failure to stop or reduce steroid dosage<br><br>*(consensus)*	
Q14.2: When treating steroid refractory cGvHD patients with ruxolitinib, what would be the reasons for stopping ruxolitinib treatment?	Yes	Average rank 1.27: Lack of efficacy<br>Average rank 1.7: Side effects<br>Average rank 2: Another reason, namely:<br>Given alternatives by experts:<br>○ Failure reduce steroid dose<br>Average rank 3: Inconvenience for the patient	Q19.2<br>Rank 1: Lack of efficacy<br>Rank 2: Side effect<br>Rank 3: Other reasons, e.g. failure to reduce steroid dosage<br><br>*(consensus)*	-
Q15.1: When treating steroid refractory aGvHD patients with combination therapy	Yes	Average rank 1.36: Lack of efficacy<br>Average rank 1.8: Side effects	Q20.1<br>Rank 1: Lack of efficacy	-

Question	Consensus	Round 1 Results	Round 2 Results	Round 3 Results
(ECP + ruxolitinib), what would be reasons to stop the combination treatment?		Average rank: 2: Another reason, namely:<br>Given alternatives by experts:<br>○ Failure to reduce steroid dose<br>Average rank 2.75: Inconvenience for the patient	Rank 2: Side effects<br>Rank 3: Other reasons, e.g. failure to reduce steroid dosage<br><br>*(consensus)*	
Q15.2: When treating steroid refractory cGvHD patients with combination treatment (ECP + ruxolitinib), what would be reasons to stop the combination treatment?	Yes	Average rank 1.27: Lack of efficacy<br>Average rank 1.8: Side effects<br>Average rank 2: another reason, namely:<br>Given alternative by experts:<br>○ Failure reduce steroid dose<br>Average rank 2.89: Inconvenience for the patient	Q20.2<br>Rank 1: Lack of efficacy<br>Rank 2: Side effects<br>Rank 3: Other reasons e.g. failure to reduce steroid dosage<br><br>*(consensus)*	-

Question	Consensus	Round 1 Results	Round 2 Results	Round 3 Results
Q16.1: Based on your experience please list the reasons you would NOT add ECP to ruxolitinib in steroid refractory aGvHD patients. Please order the reasons from most important to least important	Partial	Average rank 1.33: Inconvenience for the patient<br>Average rank 2: other reasons, please specify<br>Given alternatives by experts:<br>○ Venous access lacking of now central line<br>○ Low white blood cell counts<br>Average rank 2.2: Low efficacy<br>Average rank 2.43: High workload for the hospital staff<br>Average rank: 2.75: ECP capacity in my center is restricted	Q21.1<br>Rank 1: Inconvenience for the patient<br>Rank 2: other reasons, e.g. lack of venous access or low white blood cell count<br>Rank 3: Low efficacy<br><br>*(partial consensus)*	*(not asked in again in third round)*
Q16.2: Based on your experience please list the reasons you would NOT add ECP to	Yes	Average rank 1.27: Inconvenience for the patient	Q21.2	Q21.2

Question	Consensus	Round 1 Results	Round 2 Results	Round 3 Results
ruxolitinib in steroid refractory cGvHD patients. Please order the reasons from most important to least important.		Average rank 2.17: High workload for the hospital staff<br>Average rank 2.4: ECP capacity in my center is restricted<br>Average rank 3: Low efficacy	Rank 1: Inconvenience for the patient<br>Rank 2: High workload for the hospital staff<br>Rank 3: Restricted ECP capacity<br><br>*(partial consensus)*	Rank 1: Inconvenience for the patient<br>Rank 2: Organizational/logistic reasons (such as restricted hospital capacity)<br><br>*(consensus)*
Q17: Based on your experience and depending on the treatment schemes: When do you decide to add an additional treatment to the initial therapy?	Yes	Aggregated data:<br>Average time period from ruxolitinib to addition of ECP<br>○ aGvHD: 14 days<br>○ cGvHD: 60 days	Q22.1<br>What is the average time period until addition of ruxolitinib or ECP in aGvHD? Do you agree on these statements, resulting from round 1?	-

Question	Consensus	Round 1 Results	Round 2 Results	Round 3 Results
		Average time period from ECP to addition of ruxolitinib<br>○ aGvHD: 14 days<br>○ cGvHD: 90 days	○ The average time period from ECP to addition of ruxolitinib is 14 days in aGvHD patients<br>○ The average time period from ruxolitinib to addition of ECP is 14 days in aGvHD patients<br><br>*(consensus)*<br><br>Q22.2<br>What is the average time period until addition of ruxolitinib or	

Question	Consensus	Round 1 Results	Round 2 Results	Round 3 Results
			ECP in cGvHD? Do you agree on these statements, resulting from round 1?<br>○ The average time period from ECP to addition of ruxolitinib is 2 months in aGvHD patients<br><br>*(consensus)*	
Q18: Please list below the advantages/ of using a sequential treatment regimen of ECP and ruxolitinib - cGvHD	Yes	Please see Appendix Table 4 below.	Q23.1<br>Please list the advantages/ of using a sequential treatment regimen of ECP and ruxolitinib in	-

Question	Consensus	Round 1 Results	Round 2 Results	Round 3 Results
			aGvHD. Do you agree on this ranking, resulting from round 1?<br>Rank 1: Improved response (incl. faster and more durable response)<br>Rank 2: Safety aspects (e.g. less side effects, fewer hospitalization, no increase in toxicity)<br>Rank 3: Steroid sparing effects (e.g. incl. faster steroid tapering)<br><br>*(consensus)*	

Question	Consensus	Round 1 Results	Round 2 Results	Round 3 Results
Q18: Please list below the advantages/ of using a sequential treatment regimen of ECP and ruxolitinib - cGvHD	Yes	Please see Appendix Table 5 below.	Q23.2<br>Please list the advantages/ of using a sequential treatment regimen of ECP and ruxolitinib in cGvHD. Do you agree on this ranking, resulting from round 1?<br>Rank 1: Improved response (incl. faster and more durable response)<br>Rank 2: Safety aspects (e.g. less side effects, fewer hospitalization, no increase in toxicity)<br>Rank 3: Steroid sparing effects (incl. faster steroid tapering)	-

Question	Consensus	Round 1 Results	Round 2 Results	Round 3 Results
			(consensus)	
Q19.1: In your experience, would it be possible to taper the dosage of ruxolitinib as part of a combination therapy with ECP at all?	-	aGvHD: yes=100%<br>cGvHD: yes=100%<br><br>*(only asked in first round)*	-	-
Q19.2: In your experience, would it be possible to taper the ECP treatment schedule as part of a combination therapy with ruxolitinib at all?	-	aGvHD: yes= 82% no=18%<br>cGvHD: yes=91% no= 9%<br><br>*(only asked in first round)*	-	-
Q19.3: If steroid refractory GvHD patients respond to the combination therapy of ECP and ruxolitinib you may want to reduce treatment. How would you design the reduction of combination therapy?	Yes	aGvHD<br>○ Simultaneous reduction of ruxolitinib and ECP: 9%<br>○ Reduction of ruxolitinib first: 64%	Q24.1<br>If steroid refractory aGvHD patients respond to the combination therapy of ECP and ruxolitinib you may want to	-

Question	Consensus	Round 1 Results	Round 2 Results	Round 3 Results
		○ Reduction of ECP first: 27%	reduce treatment. How would you design the reduction of combination therapy?<br>○ When reducing combination therapy in aGvHD patients, ruxolitinib is reduced first in 64% of cases<br>○ When reducing combination therapy in aGvHD patients, ECP is reduced first in 27% of cases<br>○ When reducing combination therapy in	

Question	Consensus	Round 1 Results	Round 2 Results	Round 3 Results
			aGvHD patients, ruxolitinib and ECP are reduced simultaneously in 9% of cases<br><br>*(consensus)*	
Q19.3: If steroid refractory GvHD patients respond to the combination therapy of ECP and ruxolitinib you may want to reduce treatment. How would you design the reduction of combination therapy?	Yes	cGvHD<br>○ Simultaneous reduction of ruxolitinib and ECP: 0%<br>○ Reduction of ruxolitinib first: 73%<br>○ Reduction of ECP first: 27%	Q24.2<br>If steroid refractory cGvHD patients respond to the combination therapy of ECP and ruxolitinib you may want to reduce treatment. How would you design the reduction of combination therapy?	-

Question	Consensus	Round 1 Results	Round 2 Results	Round 3 Results
			○ When reducing combination therapy in cGvHD patients, ruxolitinib is reduced first in 73% of cases<br>○ When reducing combination therapy in cGvHD patients, ECP is reduced first in 27% of cases<br>○ When reducing combination therapy in cGvHD, there is no simultaneously	

**Question**	**Consensus**	**Round 1 Results**	**Round 2 Results**	**Round 3 Results**
			reduction of ruxolitinib<br><br>and ECP<br><br><br>*(consensus)*	
Q20: Is the ECP treatment schedule adapted when used in combination?	-	**aGvHD**<br><br>No=91%<br><br>Yes, decreasing frequency=9%<br><br><br>**cGvHD**<br><br>No=91%<br><br>Yes, decreasing frequency=9%<br><br><br>*(only asked in first round)*	-	-
Q21: Please list the 3 therapies you are most likely to combine with ECP: aGvHD	Q25.1 Partial	Please see Appendix Table 6 below.	Q25.1	25.3

Question	Consensus	Round 1 Results	Round 2 Results	Round 3 Results
	Q25.3<br>Consensus		List the 3 therapies you are most likely to combine with ECP in aGvHD. Do you agree on this ranking, resulting from round 1?<br>Rank 1: Ruxolitinib<br>Rank 2: Fecal microbiota transplantation<br>Rank 3: TNF-alpha inhibitors<br>Rank 4: mTOR inhibitors, ATG<br>Rank 5: others, e.g. Ibrutinib, Vedolizumab, alpha-1-antitrypsin, etanercept<br><br>*(partial consensus)*	In round 1 and 2, you agreed that ruxolitinib is the treatment that is most likely combined with ECP in acute GvHD. Do you have any objections combining ECP with any other guideline recommended therapy?<br><br>No obstacles reported<br><br>*(consensus)*

**Question**	**Consensus**	**Round 1 Results**	**Round 2 Results**	**Round 3 Results**
Q21: Please list the 3 therapies you are most likely to combine with ECP: cGvHD	Q25.2 Partial<br><br>Q24.5<br>Consensus	Please see Appendix Table 7 below.	25.2<br>List the 3 therapies you are most likely to combine with ECP in cGvHD. Do you agree on this ranking, resulting from round 1?<br>Rank 1: Ruxolitinib<br>Rank 2: mTOR inhibitors<br>Rank 3: Ibrutinib, belumosudil, TNF-alpha inhibitors, mycophenolate mofetil<br><br>*(partial consensus)*	25.4<br>In round 1 and 2, you agreed that ruxolitinib is the treatment that is most likely combined with ECP in chronic GvHD. Do you have any objections combining ECP with any other guideline recommended therapy?<br><br>No obstacles reported<br><br>*(consensus)*
Q22: Based on your experience, what would be the hurdles preventing a timely use of	Yes	Average rank 1.73: Difficulties with venous access for ECP	Q26	-

Question	Consensus	Round 1 Results	Round 2 Results	Round 3 Results
ECP after steroid refractoriness has been established? Please rank the following reasons from most important to least important.		Average rank 1.86: Restricted capacity of the ECP unit<br>Average rank 3: Patient decision<br>Average rank 3: restricted hospital bed capacity	What would be the hurdles preventing a timely use of ECP after steroid refractoriness has been established?<br>Rank 1: Difficulties with venous access for ECP<br>Rank 2: Restricted capacity of ECP unit<br>Rank 3: Patient decision<br><br>*(consensus)*	
Q23: Do you see the potential of using ECP as a monotherapy (without steroids) in GvHD patients?	Yes	**aGvHD**<br>Yes=73%<br>No=27%	Q27<br>Do you see the potential of using ECP as a monotherapy	-

Question	Consensus	Round 1 Results	Round 2 Results	Round 3 Results
		**cGvHD**<br>Yes=91%<br>No=9%	treatment (without steroids) in aGvHD patients?<br>ECP shows the potential to be used as a monotherapy (without steroids) in aGvHD patients<br><br>*(consensus)*	
-	Yes	-	Q28.1/Q28.2 In the first round of questioning, you indicated that you see the potential for ECP monotherapy (without steroids) in cGvHD patients (91% consensus among participants). Please list the selection criteria to treat aGvHD	Q28<br>Do you agree with the results from round 2 on the selection criteria for treating steroid-refractory GvHD (both acute and chronic) patients with ECP monotherapy?

Question	Consensus	Round 1 Results	Round 2 Results	Round 3 Results
			patients with ECP monotherapy.<br>Q28.1 Please see Appendix Table 8 below<br>Q28.2 Please see Appendix Table 9 below	Rank 1: Low risk (e.g. skin involvement only or upper GI only<br>Rank 2: contraindication for Ruxolitinib (e.g. thrombocytopenia)<br><br>*(consensus)*
Q24: What percentage of GvHD patients would you consider treating with ECP even if they are not refractory to steroids?	Yes	aGvHD: 36 patients<br>cGvHD: 27 patients	Q29.1<br>What percentage of aGvHD patients would you consider treating with ECP even if they are not refractory to steroids? Do you agree on this statement, resulting from round 1?	-

Question	Consensus	Round 1 Results	Round 2 Results	Round 3 Results
			In approx. 25% of aGvHD patients, ECP is considered even if they are not steroid refractory<br><br>*(consensus)*<br><br>Q29.2<br>What percentage of cGvHD patients would you consider treating with ECP even if they are not refractory to steroids? Do you agree on this statement, resulting from round 1?	

Question	Consensus	Round 1 Results	Round 2 Results	Round 3 Results
			In approx. 27% of cGvHD patients, ECP is considered even if they are not steroid refractory<br><br>*(consensus)*	
Q25: You have now reached the end of the questionnaire. Are there any other aspects related to ECP treatment of steroid-refractory GvHD patients that are of high importance to you and were not addressed in the questionnaire?	-	The following aspects were mentioned by the experts:<br>○ Which are the most common adverse events of combination treatment? How are patients with thrombocytopenia managed?<br>○ Reasons for combination therapy of ECP and any other agent as first line	-	-

Question	Consensus	Round 1 Results	Round 2 Results	Round 3 Results
		treatment of steroids refractory acute or chronic GvHD<br>○ Infectious complications and immune reconstitution		
-	-	-	-	Q30.1: Which data generation strategy would you recommend Therakos/Mallinckrodt pursues in aGvHD? Please rank the suggestions below:<br>Average rank 1.45: Prospective study in steroid - refractory aGvHD

Question	Consensus	Round 1 Results	Round 2 Results	Round 3 Results
				combination ECP and ruxolitinib<br>Average rank 2.18: Early use of ECP as monotherapy or in combination with steroids<br>Average rank 2.55: Retrospective study on steroid-refractory aGvHD combination ECP and ruxolitinib<br>Average rank 3.18: Prospective study for efficacy of ECP in steroid-refractory aGvHD

Question	Consensus	Round 1 Results	Round 2 Results	Round 3 Results
				Average rank 3.73: Proof of concept of ECP in prophylaxis
-	-	-	-	Q30.2: Which data generation strategy would you recommend Therakos/Mallinckrodt pursues in cGvHD? Please rank the suggestions below<br>Average rank 1.64: Prospective study in steroid - refractory cGvHD combination ECP and ruxolitinib<br>Average rank 2.27: Early use of ECP as monotherapy or in combination with steroids

Question	Consensus	Round 1 Results	Round 2 Results	Round 3 Results
				Average rank 2.45: Prospective study in steroid-refractory cGvHD combination ECP and belumosudil<br>Average rank 2.91: Retrospective study in steroid-refractory cGvHD combination ECP and ruxolitinib<br>Average rank 3.82: Prospective study for ECP in steroid-refractory cGvHD (single arm)

Abbreviations: aGvHD: acute graft-versus-host disease; ATG: autophagy-related gene; cGvHD: chronic graft-versus-host disease; CNI: calcineurin inhibitor; ECP: extracorporeal photopheresis; EMA: European Medicines Agency; GvHD: graft-versus-host disease; IS: immunosuppression; mTOR: mammalian target of rapamycin; PR: partial response; SR: steroid-refractory; TNF-alpha: Tumor Necrosis Factor alpha.

Appendix Table 4: Experts' main reasons for choosing treatments for patients with aGvHD

ECP but no ruxolitinib	Ruxolitinib but no ECP	ECP & ruxolitinib together
Safety profile, contraindication to ruxolitinib	Efficacy	Efficacy
High efficacy, no toxicity, no severe cytopenias	No need for central venous catheter, prompt response, steroid-sparing	In patients with severe gi gvhd to improve response
Thrombocytopenia; DILI (liver damage)	Difficult vascular access; patient ECOG status	Very aggressive disease
Efficacy in grade 2-3 skin or mucosal aGVHD	Efficacy in grade 2-4 or intestinal aGVHD	Efficacy in grade 2-4 intestinal, skin, hepatic or lung aGVHD
Contraindication for ruxolitinib	Ema approved drug for this indication	Synergistic effect
Skin	Gastrointestinal	Severe grade iv aGVHD early after transplantation
Contraindication to ruxolitinib (low platelets), or ruxo failure	Ruxo is now considered as the main second line in steroid resistant patients in our center	Partial response to ruxo, especially in case of skin aGVHD
Cytopenia and active viral infections	First line treatment in the absence of severe cytopenia or viral infection. Heart failure would be for me a indicator to prefere another treatment instead of ecp	Severity grade of GvHD

Efficacy, safety	Published data	Rarely
Infections, cytopenia	Higher response rate, faster response	Higher efficacy without increased infectious risks
Especially in skin involvement, Failure / adverse events (ie thrombotic microangiopathy) of ruxolitinib	Patient preference, venous access, machine availability	Acute severe cases

Abbreviations: aGVHD: acute graft-versus-host disease**;** DILI: drug induced liver injury; ECOG: Eastern Cooperative Oncology Group**;** ECP: extracorporeal photopheresis; IV: intravenous.

Appendix Table 5: Experts' main reasons for choosing treatments for patients with cGvHD

ECP but no ruxolitinib	Ruxolitinib but no ECP	ECP & ruxolitinib together
Safety profile, contraindication to ruxolitinib	Efficacy	Efficacy
High efficacy, excellent safety profile, tolerated well	Good patient adherance, prompt response, well tolerated	Not done
Poor hematological tolerance; poor liver function	Patient compliance & difficult vascular access	Multiorgan involvement
Efficacy in moderate cgvhd	Efficacy in moderate or severe hepatic or lung cGVHD	Efficacy in moderate or severe hepatic or lung cGVHD
Contraindication for ruxolitinib e.g. Cytopenia	EMA approved drug for this indication	Synergistic effect
Skin	Gastrointestinal	Partial remission with eco or ruxo only
Contraindication to ruxolitinib (low platelets), or ruxo failure	Ruxo is now considered as the main second line in steroid resistant patients in our center	Partial response to ruxo, especially in case of skin/mucosal cGVHD
Cytopenia and active viral infections	First line treatment in the absence of severe cytopenia or viral infection. Heart failure would be for me a indicator to prefere another treatment instead of ecp. Compliance	Severity grade of GvHD

Efficacy, steroid sparing	Published data	Rarely
Infections, cytopenia, relapse risk	Higher response rate, oral drug, approval	Higher response rate without increased infectious risks
Especially in skin involvement, Failure / adverse events (ie thrombotic microangiopathy) of ruxolitinib	Patient preference, venous access, machine availability	Severe cases

Abbreviations: cGvHD: chronic graft-versus-host disease**;** ECP: extracorporeal photopheresis; EMA: European Medicines Agency; GvHD: graft-versus-host disease.

Appendix Table 6: List and rank of the advantages of using the sequential treatment regimen of ECP and ruxolitinib in patients with aGvHD

Advantage 1	Advantage 2	Advantage 3
Improve response rate	Safety	
No advantage	No advantage	No advantage
Improving response rate	Accelerate steroid sparing	Avoid Ruxo in ECP-sensitive patients
Low toxicity of ECP	Higher efficacy	Steroids sparing
Some patients may respond to the monotherapy	Less hospitalization if the patient responds to ruxo only	Potentially less side effects
Improved response rate	Faster steroid tapering	
Steroid-sparing effect	Deeply and durable response	Limitation of side effects of both treatments if given for a shorter period due to increased and rapid efficacy of the combination
we do not usuially use ruxo and ECP in sequence	I suppose it may increase efficacy	I suppose it may further reduce steroid therapy
faster response	higher response rate	no increase in toxicity
Reduction of side effects	Clear effect of each treatment	Long-term efficacy

Footnotes: Responses were only obtained for 10 out of 11 experts. One expert did not provide a response.

Abbreviations: aGvHD: acute graft-versus-host disease; ECP: extracorporeal photopheresis.

Appendix Table 7: List and rank of the advantages of using the sequential treatment regimen of ECP and ruxolitinib in patients with cGvHD

Advantage 1	Advantage 2	Advantage 3
Improve efficacy	Safety of ECP	
Lower steroid dose at start of ruxo	No infections at start of ruxo	
Improving the response rate	Accelerate and widen steroid sparing	Avoid Ruxo in ECP-sensitive patients
Low toxicity of ECP	Higher efficacy	Steroids sparing
Some patients may respond to the monotherapy	Less hospitalization if the patient responds to ruxo only	Potentially less side effects
Improved response rate	Faster steroid tapering	
Steroid-sparing effect	Deeply and durable response	Limitation of side effects of both treatments if given for a shorter period due to increased and rapid efficacy of the combination
We do not usually use ruxop and ECP in sequence	I suppose it may increase efficacy	I suppose it may further reduce steroid therapy
Higher response rate	No increase in toxicity	No increase in toxicity
Reduction of side effects	Clear effect of each treatment	Long-term efficacy

Footnotes: Responses were only obtained for 10 out of 11 experts. One expert did not provide a response.

Abbreviations: cGvHD: chronic graft-versus-host disease; ECP: extracorporeal photopheresis.

Appendix Table 8: List of 3 therapies most likely combined with ECP in patients with aGvHD

Therapy 1	Therapy 2	Therapy 3
Steroids	Ruxolitinib	Fecal microbiota transfer
Ruxolitinib	Fecal microbiota transplant	Alpha-1-antitrypsin
Steroids	Ruxolitinib	Vodolizumab
Ruxolitinib	MMF	Infliximab
Ruxolitinib	Everolimus	Infliximab
Ruxolitinib	Fecal microbiota transplantation	
Ruxolitinib	Etanercept	Atg
Steroid + CNI	Ruxolitinib	TNF-alpha inhibition
Steroids	Cyclosporin	Mycophernolate
Ruxolitinib	ATG	Tacrolimus
Steroids	Ruxolitinib	Ibrutinib

Abbreviations: aGvHD: acute graft-versus-host disease; ATG: autophagy-related gene; CNI: calcineurin inhibitor; ECP: extracorporeal photopheresis; MMF: mycophenolate mofetil; TNF-alpha: Tumor Necrosis Factor alpha.

Appendix Table 9: List of 3 therapies most likely combined with ECP in patients with cGvHD

Therapy 1	Therapy 2	Therapy 3
Steroid	Ruxolitinib	Belumosudil
Ruxolitinib	Sirolimus	Everolimus
Ruxolitinib	Imatinib	Rituximab
Ruxolitinib	MMF	Sirolimus
Ruxolitinib	Everolimus	Mmf
Imatinib	Ibrutinib	Ruxolitinib
Ruxolitinib	Belumosudil	Ibrutinib
Steroid + CNI	Ruxolitinib	TNF-alpha inhibition and/or Belumosudil
Steroids	Cyclosporin	Mycophenolate
Ruxolitinib	Everolimus	Tacrolimus
Steroids	Ruxolitinib	Ibrutinib

Abbreviations: cGvHD: chronic graft-versus-host disease; CNI: calcineurin inhibitor; ECP: extracorporeal photopheresis; MMF: mycophenolate mofetil; TNF-alpha: Tumor Necrosis Factor alpha.

Appendix Table 10: Selection criteria listed by the experts for the treatment of patients with aGvHD with ECP monotherapy

	**Criterion 1**	**Criterion 2**	**Criterion 3**	**Criterion 4**	**Criterion 5**
Expert 1	Early diagnosis	Skin	Upper GI	Minimal lower GI	Early liver
Expert 2	Skin involvement	Thrombocytopenia	-	-	-
Expert 3	Low risk aGvHD (low biomarker score)	-	-	-	-
Expert 4	Contraindication for ruxo	-	-	-	-
Expert 5	Stage 1-2 skin aGvHD	-	-	-	-
Expert 6	Low toxicity	-	-	-	-
Expert 7	Patients with acute GvHDAnn Arbor score 1 (low risk acc. to MAGIC criteria)	-	-	-	-
Expert 8	Mild aGvHD	-	-	-	-
Expert 9	Skin involvement	-	-	-	-
Expert 10	Skin involvement	Mild GUT manifestation	-	-	-

Footnotes: Responses were only obtained for 10 out of 11 experts. One expert did not provide a response.

Abbreviations: aGvHD: acute graft-versus-host disease; ECP: extracorporeal photopheresis; GI: gastrointestinal; GvHD: graft-versus-host disease; MAGIC: Mount Sinai Acute GvHD International Consortium.

Appendix Table 11: Selection criteria listed by the experts for the treatment of patients with cGvHD with ECP monotherapy

	**Criterion 1**	**Criterion 2**	**Criterion 3**
Expert 1	Early diagnosis	Pre-empitve on day +100)	-
Expert 2	Skin involvement	Thrombocytopenia	-
Expert 3	cGvHD skin or mucosal limited	Not overlap syndrome	-
Expert 4	Low risk moderate cGvHD	-	-
Expert 5	Venous access	Hospitalized	-
Expert 6	Mild cGvHD with only skin or mucosal involvement	-	-
Expert 7	Low toxicity	-	-
Expert 8	Patients with standard risk and moderate involvement of one or two organs	-	-
Expert 9	Mild cGvHD with involvement of skin and/or gut and/or mucosa	-	-
Expert 10	Skin involvement	-	-
Expert 11	Superficial prevalent skin lesions	Oral mucose involvement	Eye involvement

Abbreviations: cGvHD: chronic graft-versus-host disease; ECP: extracorporeal photopheresis.
